# N-Acetylglucosamine Kinase, *HXK1* Is Involved in Morphogenetic Transition and Metabolic Gene Expression in *Candida albicans*

**DOI:** 10.1371/journal.pone.0053638

**Published:** 2013-01-14

**Authors:** Kongara Hanumantha Rao, Swagata Ghosh, Krishnamurthy Natarajan, Asis Datta

**Affiliations:** 1 National Institute of Plant Genome Research, New Delhi, India; 2 The Laboratory of Eukaryotic Gene Expression, School of Life Sciences, Jawaharlal Nehru University, New Delhi, India; New Jersey Medical School, University of Medicine and Dentistry of New Jersey, United States of America

## Abstract

*Candida albicans,* a common fungal pathogen which diverged from the baker’s yeast *Saccharomyces cerevisiae* has the unique ability to utilise N-acetylglucosamine, an amino sugar and exhibits phenotypic differences. It has acquired intricate regulatory mechanisms at different levels in accordance with its life style. N-acetylglucosamine kinase, a component of the N-acetylglucosamine catabolic cascade is an understudied gene since *Saccharomyces cerevisiae* lacks it. We report *HXK1* to act as both positive and negative regulator of transcription of genes involved in maintaining cellular homeostasis. It is involved in repression of hyphal specific genes in addition to metabolic genes. Its regulation of filamentation and GlcNAc metabolism is independent of the known classical regulators like *EFG1*, *CPH1*, *RAS1*, *TPK2* or *TUP1*. Moreover, Hxk1-GFP is localised to cytoplasm, nucleus and mitochondria in a condition specific manner. By employing two-step affinity purification, we report the interaction of *HXK1* with *SIR2* under filamentation inducing conditions. Our work highlights a novel regulatory mechanism involved in filamentation repression and attempts to decipher the GlcNAc catabolic regulatory cascade in eukaryotes.

## Introduction

The human pathogen *Candida albicans* resides commensally in most healthy individuals, but causes severe infections in immuno-compromised patients. This pathogen has gained the ability to utilise diverse carbon sources available in the host environment. One of the reasons for virulence has been assigned to its ability of switching between yeast and hyphal form triggered by various environmental cues including the presence of N-acetylglucosamine (GlcNAc) [1], [2], [3]. The pathways that are involved in morphogenetic switching in Candida are under the control of several signaling pathways and transcription factors [4], [5]. The most prominent positive regulators include a mitogen activated protein (MAP) kinase pathway and its downstream transcription factor Cph1/Acpr [6], [7], as well as the cyclic adenosine mono-phosphate (cAMP)/protein kinase (PKA) pathway and its downstream target transcription factor Efg1 [8], [9]. In addition, filamentation is also under the negative control of a transcriptional repressor Tup1 [10].

Although previous reports on N-acetylglucosamine inducible genes have highlighted GlcNAc as a structurally and functionally important molecule, its role in cellular signaling is not yet fully understood [11], [12], [13]. The phosphorylation of this signalling molecule by *HXK1* probably triggers the GlcNAc signaling cascade. In eukaryotes as such, the evidences of metabolic enzymes taking part in signal transduction pathways are relatively few [14], [15]. Moreover much of the focus till date has been on the Galactose regulatory circuit, whereas the GlcNAc signaling pathway which is well conserved among the pathogenic and non-pathogenic organisms (fungi and other eukaryotes) [16], [12] is poorly defined, until a recent study showed that GlcNAc entering the cell is sufficient to induce the signaling irrespective of GlcNAc being catabolised [17]. Thus it is important to study the regulation for GlcNAc metabolic gene expression to understand the evolutionary aspects of rewiring adopted to survive under various carbon sources.

Our work emphasises on characterization of hexokinase enzyme from *Candida albicans*, known to be the most evolutionarily conserved sugar sensor across the species of yeast, mammals and plants [18], [19], [20], [21]. In this study we propose *HXK1* to play a crucial role in morphogenesis, GlcNAc signaling/metabolic genes expression and maintenance of various cellular functions in *Candida albicans*. In correlation with its function of filamentation repression we report the interaction of this enzyme with a histone deacetylase that could be instrumental in maintaining the repressed state of some Hyphal specific genes (HSGs).

## Results

### HXK1 Mutant is Constitutively Filamentous and Hyperfilamentous in Filamentation Inducing Conditions

**Figure 1:**
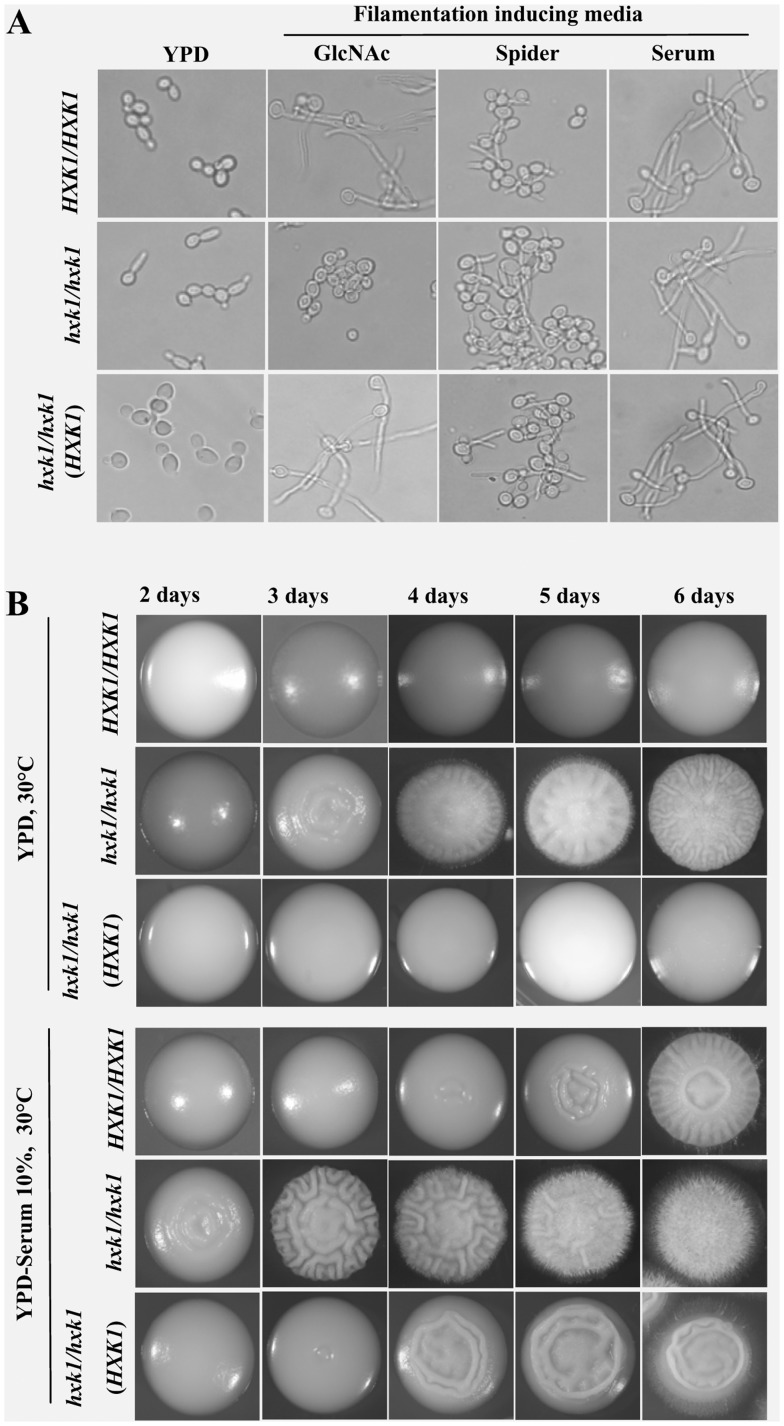
*hxk1* mutant is constitutively filamentous and hyperfilamentous in filamentation inducing conditions. A) *hxk1* mutant is hyperfilamentous in liquid filamentation inducing media like Spider and Serum as compared to wild type strain (CAF2–1) and showed germ tube like protuberances (28–35%) in YPD liquid medium grown at 30°C (details are given in Text S1). B) *hxk1* mutant is hyper filamentous and showed filamentation early in YPD and YPD+serum solid plates at 30°C after 3 and 2 days respectively whereas wild type and revertant colonies grown under similar conditions were completely smooth.

Singh *et al.* (2001) observed that *C. albicans* mutant with disruption in the *N*-acetylglucosamine (GlcNAc) catabolic pathway gene cluster (*nag cluster* mutant), including the GlcNAc-6-phosphate deacetylase (*dac1*), glucosamine-6-phosphate deaminase (*nag1*), and GlcNAc kinase (*hxk1*) genes, was not able to grow on amino sugars, exhibited highly attenuated virulence in a murine systemic candidiasis model but showed hyperfilamentation under stress-induced filamentation conditions. Such hyperfilamentation has also been reported in an *hxk1*mutant by a separate group [22]. Here, we have isolated and characterized in detail, an *hxk1* mutant that showed hyperfilamentation similar to that of *nag* cluster mutant. A homozygous *hxk1* mutant (H8–1–103) under non-inducing growth conditions showed germ tube like protuberances (28–35%) in YPD liquid medium whereas the wild type cells under similar conditions were completely in the blastospore form (Figure. 1A). Furthermore, in YPD and YPD+serum solid plates at 30°C after 3 and 2 days respectively the homozygous mutant started to show wrinkled colony morphology whereas wild type colonies grown under similar conditions were completely smooth (Figure. 1B). The appearance of filaments started much earlier in the mutant when compared to the wild type on solid plates. In order to study the degree of hyperfilamentation during inducing conditions in the mutant we chose Spider liquid medium where 50% of the wild type cells tend to form filaments. Under such a condition the effect of the mutation of this gene could be observed more clearly (Figure 1A). Within 1–2 hours of induction of the mutant in this medium almost all cells were either filamentous or at least showed germ-tubes. In presence of serum liquid medium (serum is a strong inducer of filamentation), no clear differences were observed whereas on solid serum plates homozygous mutants showed hyperfilamentation. These results overall show that the *hxk1*mutant was somewhat predisposed to filamentous growth and thus showed high degree of filamentation in inducing conditions and filamented even under non-inducing conditions. The failure of *hxk1* mutant to form germ tubes in response to GlcNAc salt based medium was due to its inability to catabolise the sugar, therefore, when the medium was supplemented with glucose or in the case of revertant, the filamentation was more than that of wild type (Figure 1A).

### HXK1 Expression Levels are Upregulated in Filamentation Inducing Media

**Figure 2:**
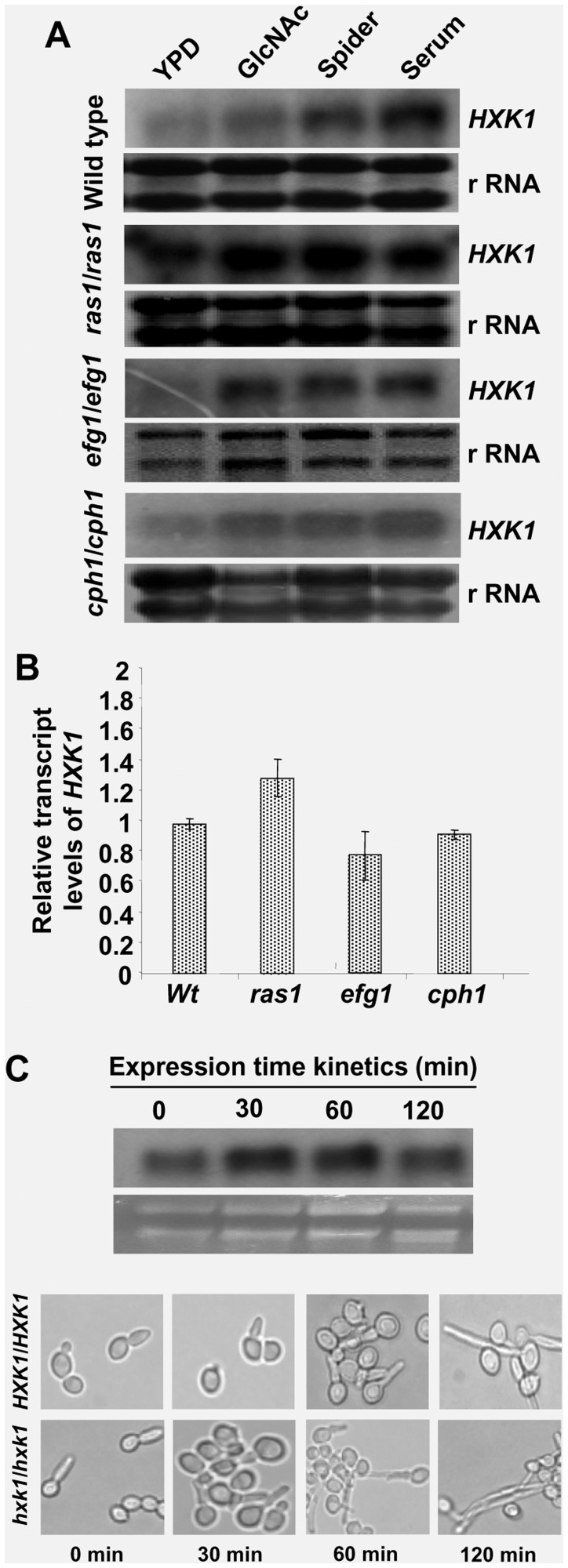
Expression pattern of *HXK1* in filamentation inducing media. A) Hxk1 transcript levels are upregulated in response to filamentation conditions in wild type and mutants for filamentation regulators. Wild-type strain and mutants were grown in YPD for 6 hr at 30°, washed, starved for 10 hrs and induced in YPD, GlcNAc, Spider and Serum (Experimental procedures). Cells were harvested at 2 hrs time point in case of YPD, Spider or Serum and at 4 hrs time point in case of GlcNAc and the total RNA was prepared and northern blot analyses were carried out. Ribosomal (r RNA) has been included as loading control. B) Quantitative RT-PCR of *HXK1* basal transcript levels for the wild type and mutant cells grown in YPD. Almost unaltered HXK1 mRNA levels were observed in all strains. Error bars represent the coefficient of variation (n = 3). C) Northern blot analysis for the time kinetics of *HXK1.* Transscript levels are in response to Spider at 37°C. Ribosomal (r RNA) has been included as loading control. Corresponding cell shots representing hyperfilamentous morphology of *hxk1* mutant in comparison with wild type strain have also been shown at the bottom.

Earlier reports from our lab [23] clearly showed the up-regulation of *HXK1* under conditions of GlcNAc catabolism where the aspect of filamentation was not dealt with. To investigate the aspect of regulation of filamentation by *HXK1*, we checked the transcript levels of *HXK1*during filamentation inducing conditions like GlcNAc (YNB-salt base with 2.5 mM GlcNAc) [24], Spider and 20% serum and compared with non-inducing conditions in wild type and filamentation specific pathway mutants, *ras1*(CAN50), *efg1*(HLC52) and *cph1*(A11–1). In all filamentation inducing media, in the mutants (*ras1*, *efg1* and *cph1*), *HXK1* levels were upregulated similar to that of wild type (Figure. 2A) even though the mutants failed to form filaments. Since the blots are different for wild type and mutants, we have checked the relative basal transcript levels of *HXK1* in the three mutants for the cells grown in YPD which were almost comparable with the wild type (Figure 2B). The upregulated expression pattern of *HXK1* in filamentation inducing media probably supported its key role in the filamentation process. Our Northern analyses also suggested that *HXK1* induction was independent of *RAS1*, *EFG1* and *CPH1* pathways. A time kinetic analysis for *HXK1* in Spider medium showed that once induced, the transcript levels almost maintained a steady state. (Figure 2C).

### HXK1 Mediated Filamentation Works Independent of RAS1, CPH1, EFG1 or TPK2 Pathways

**Figure 3:**
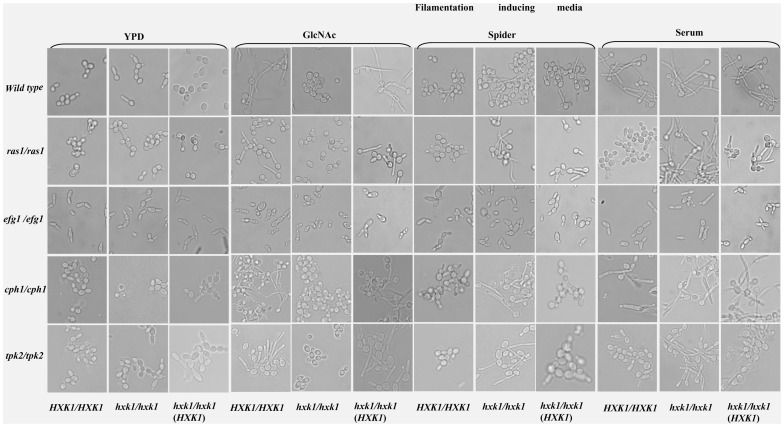
Morphological and expression pattern analyses among wild type, *hxk1* and different filamentation pathway mutants. A) Morphology of wild type, filamentation pathway mutants under different hypha-inducing conditions. The starved cells of wild type (*HXK1*/*HXK1* ) and *hxk1* mutant*(hxk1/hxk1), ras1/ras1, ras1/ras1 hxk1/hxk1, efg1/efg1, efg1/efg1 hxk1/hxk1, cph1/cph1, cph1/cph1 hxk1/hxk1, tpk2/tpk2, tpk2/tpk2 hxk1/hxk1l* and their respective complemented strains in which one functional copy of *HXK1* is reintroduced in the native locus were resuspended in non-inducing and various induction media such as 2.5 mM GlcNAc in salt base, Spider pH 7.2 and Serum (20%). Cells were induced at 37°C for 4 hours in case of GlcNAc, for 2 hours at 37°C in case of Spider and Serum and in case of YPD for 2 hours at 30°C. *hxk1* mutants except *efg1/efg1 hxk1/hxk1* were hyperfilamentous in filamentation inducing conditions like Spider and Serum. In response to GlcNAc, all *hxk1* mutants failed to form germ tubes due to its inability to catabolize the sugar, but the complemented strains were hyper filamentous. B) Expression of hyphal specific genes in different filamentation inducing media in filamentation pathway mutants. Northern blot analysis to show the expression of Hyphal Specific Genes (HSGs) like *HWP1, ECE1* or *RBT4* for the cells grown in various filamentation inducing media. Total RNA was isolated from wild type, CAF2–1 (*HXK1*+/*HXK1* ) and *hxk1* mutant (*hxk1/hxk1*), *ras1/ras1, ras1/ras1 hxk1/hxk1, efg1/efg1, efg1/efg1 hxk1/hxk1, cph1/cph1, cph1/cph1 hxk1/hxk1, tpk2/tpk2, tpk2/tpk2 hxk1/hxk1* strains induced in YPD, GlcNAc, Spider and 20% Serum. 20 µg of RNA was loaded in each lane and a Northern blotting was performed. As an internal control methylene blue staining of rRNA was used to ensure equal loading of RNA. *hxk1* mutants except *efg1/efg1 hxk1/hxk1* showed upregulation of HSGs in filamentation inducing conditions and upregulation was more prominent in Spider medium. C) Quantification of *HXK1* mediated filamentation : q-RT PCR analysis of HSGs (*ECE1*, *HWP1*and *RBT4*) in *hxk1* single and double mutants (*ras1 hxk1*, *efg1hxk1*, *cph1hxk1* and *tpk2 hxk1*) in Spider grown cells compared to their wild type and respective single mutants (*ras1*, *efg1*, *cph1* and *tpk2*). Comparative expression levels of HSGs for cells grown in YPD have also been shown. *ACT1* has been selected as the endogenous control. The error bars represent co-efficient of variation.

Fine tuning of the morphological response possibly is achieved through a co-ordinated regulation of both the MAP kinase and the cAMP-dependent signalling pathways in *Candida albicans* [5], [25]. To explore the relationship between *HXK1* and other known morphogenetic regulators like *RAS1*, *CPH1, EFG1* and *TPK2*, double mutants of *hxk1* were created under these backgrounds. All the strains were induced for filamentation in liquid media like GlcNAc, Spider and Serum and morphological studies along with northern blot analyses were carried out. Except *hxk1/efg1* double mutant (HLC-67–16–1–9), all other *hxk1* single and double mutants showed hyperfilamentation (Figure 3A) (irrespective of hyphae/pseudohyphae; Figure S3) which was further supported with northern blot analyses for the Hyphal Specific Genes (HSGs) like *HWP1*, *ECE1* and *RBT4* (Figure 3B). The de-repression of HSGs was prominent in *ras1/ras1hxk1/hxk1* (HR–1–4–2)*; cph1cph1/hxk1 hxk1* (AN–8–16) and *tpk2tpk2/hxk1 hxk1* (AS–1–3–1–8) double mutants in response to Spider and Serum. Further our comparative qRT- PCR analysis of HSGs (*ECE1*, *HWP1*and *RBT4*) in wild type (grown in YPD), *hxk1* single and double mutants (*ras1*, *efg1*, *cph1* and *tpk2*) along with respective single mutants *ras1*, *efg1*, *cph1* and *tpk2* (grown in Spider) revealed the contribution of *HXK1* towards filamentation (Figure 3C). In specific, the comparative transcriptome analysis among various single and double mutants elucidated the role of *HXK1* in hyperfilamentation. Thus, *hxk1* mutant showed derepression of *HWP1* and *ECE1* even though *RBT4* levels were almost same when compared with wild type, whereas in other *hxk1* double mutants (*ras1hxk1*, *cph1hxk1* and *tpk2hxk1*), the levels all HSGs were derepressed/up-regulated compared with their respective single mutants (*ras1*, *cph1* and *tpk2*). Moreover, in these double mutants the fold change for the HSGs were more when compared with fold change in between *hxk1* sole mutant and wild type. In case of *efg1* and *efg1hxk1* mutants the expression levels of HSGs were almost same which could be possibly due to *EFG1* being the major regulator of filamentation under most conditions checked.

This issue of hyperfilamentation can also be explained by the tendency to show filamentation which was supported by quantitative assessment in terms of percentage of cells that showed germ tubes/filaments after calcofluor staining (Figure S3, Table S1). The increase in percentage of cells forming filaments in *hxk1*sole mutant, various single and double mutants, thus point towards a contribution of *HXK1* in filamentation.

**Figure 4:**
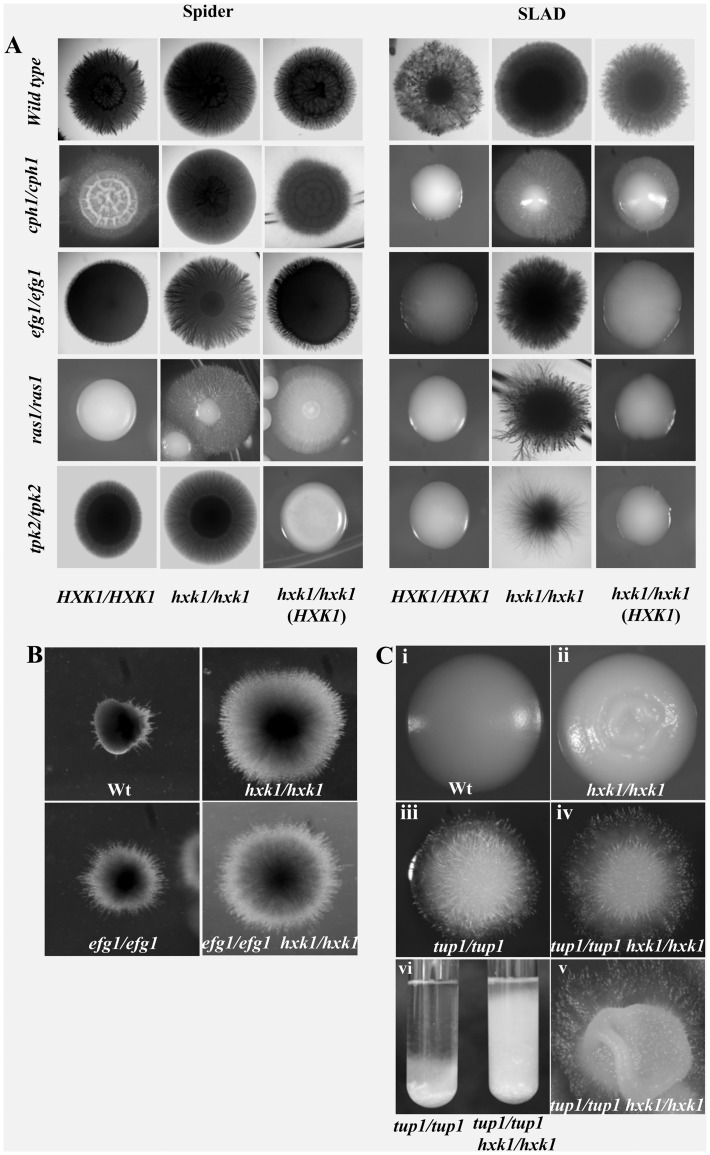
*hxk1* mutants are hyperfilamentous on filamentation inducing solid media. A) Hyphae formation on Spider and SLAD plates. All hxk1 mutants including *efg1*/*hxk1* showed hyperfilamentation. Strains were incubated at 37°C for 7 days in case of spider and 10 days in case of SLAD plates. Wild-type CAF2–1 (*HXK1*/*HXK1*); *hxk1* mutant, H8–1–103 (*hxk1/hxk1*); *cph1* mutant, A11–1 (*cph1/cph1*); double mutant of *cph1*/*hxk1,* AN8–1–16 (*cph1/cph1 hxk1/hxk1*); *efg1* mutant, HLC52 (*efg1/efg1*); double mutant of *efg1*/*hxk1* HLC67–16–1–9 (*efg1/efg1 hxk1/hxk1*); *tpk2* mutant, TPO7.4 (*tpk2/tpk2*); double mutant of *tpk2*/*hxk1*, AS1–3–1–8 (*tpk2/tpk2 hxk1/hxk1*) and their respective complemented strains in which one functional copy of *HXK1* was reintroduced in the native locus. B) *efg1/efg1 hxk1/hxk1* double mutant showed hyperfilamentation under embedded conditions**.** Cells of wild-type, CAF2–1, *hxk1/hxk1, efg1/efg1, efg1/efg1hxk1/hxk1,* mutants were grown in YPD for 5 hrs at 30°, washed in sterile water and mixed with molten CM Agar (with 1% Tween-80) plated and grown for 3 days at 25°C. *efg1 hxk1* double mutant showed hyperfilamentation when compared to *hxk1* or *efg1* single mutants (*EFG1* is reported to be a negative regulator of filamentation under micro-aerophillic/embedded conditions). C) *tup1*/*hxk1* double mutant showed growth pattern slightly different from *tup1* mutant. Wild type (i), *hxk1* mutant (ii), *tup1*(iii) and *tup1*/*hxk1*(iv) mutants colonies were grown on YPD solid and liquid medium for 3 and 2 days respectively at 30°C. *tup1/hxk1*double mutant shared most of the features with a *tup1* single mutant (iii, iv) on YPD plates, but in some colonies of the double mutant a wavy, afilamentous fringe could be observed at the centre(v). In YPD broth the *tup1* single mutant grew in clumps having the tendency to settle at the bottom, the *tup1/hxk1* double mutant showed uniform turbidity throughout the culture with clumps in the bottom (vi).

The hyperfilamentous phenotypes were more prominent in all the double mutants (*ras1/ras1hxk1/hxk1; cph1cph1/hxk1 hxk1* and *tpk2tpk2/hxk1 hxk1*) when grown on Spider and SLAD plates for 7 and 10 days respectively at 37°C (Figure 4A). So, we concluded that *HXK1* mediated filamentation process possibly worked independent of *RAS1*, *CPH1* or *TPK2* pathways. But deletion of both *EFG1* and *HXK1* resulted in morphology similar to that of *efg1* mutant alone in liquid inducing media where cells were locked in blastospore or yeast form (Figure 3A). This suggested that deletion of *HXK1* could not restore filamentation in an *efg1* mutant like that of *ras1*, *cph1* or *tpk2* mutants in liquid conditions. But in solid Spider, SLAD and matrix embedded conditions (Cornmeal agar with Tween-80) (Figure 4B) [26] the *efg1/efg1hxk1/hxk1* mutant was hyperfilamentous. Thus *HXK1* appeared to work independent of *EFG1* pathway too with the *HXK1* dependent filamentation probably being quite unique.

Overall, the process of filamentation in *Candida albicans* involves a complex regulatory network whereby the functioning of the pathways cannot be entirely separated.

### Repression of Hyphal Specific Genes by HXK1 and TUP1 may not be in a Dependent Linear Manner

The *tup1tup1/hxk1hxk1*double mutant (BCa2–9–H1) shared most of the features with a *tup1* (BCa2–10) single mutant under non-inducing conditions (Figures 4C iii and iv), but in some colonies of the double mutant a wavy, afilamentous fringe could be observed at the centre (Figure 4C v). The difference in the behaviour of the two mutants was more prominent when grown in YPD broth. While the *tup1* single mutant grew in clumps having the tendency to settle at the bottom, the *tup1/hxk1* double mutant showed uniform turbidity throughout the culture with clumps in the bottom (Figure 4C vi).

In *C. albicans* the principal role of *TUP1* is the regulation of filamentous growth through some of the cell surface genes like *HWP1, RBT4, RBT5* and *WAP1* [27]. An *hxk1* mutant showed derepression of hyphal specific genes (HSGs like *HWP1* and *RBT4* under non-inducing condition like YPD (Figure 3B). Similar kind of derepression of some of the cell surface genes was quite prominent in *hxk1* mutants created in the background of wild type, *ras1, cph1or tpk2* mutants too. Thus *HXK1* and *TUP1* seemed to repress overlapping HSGs, as a result of which the *tup1* single mutant and the *tup1/hxk1* double mutant had most of the features in common. *HXK1* functioning probably through a novel pathway, some of whose components might be uncovered by *TUP1* repression also arose based on the difference in colony morphology. Similar approaches using strains lacking each combination of *TUP1*, *CPH1*and *EFG1* followed by morphological studies was used to conclude that these three genes made independent and additive contribution towards filamentation in *C.albicans* [28]. However, a comparative transcriptome analysis under filamentation inducing condition between *hxk1* and *tup1* mutants would delineate the overlap or deviation between these two regulators of filamentation.

### Hxk1p Interacts with Histone Deacetylase Under Filamentation Inducing Conditions

**Figure 5:**
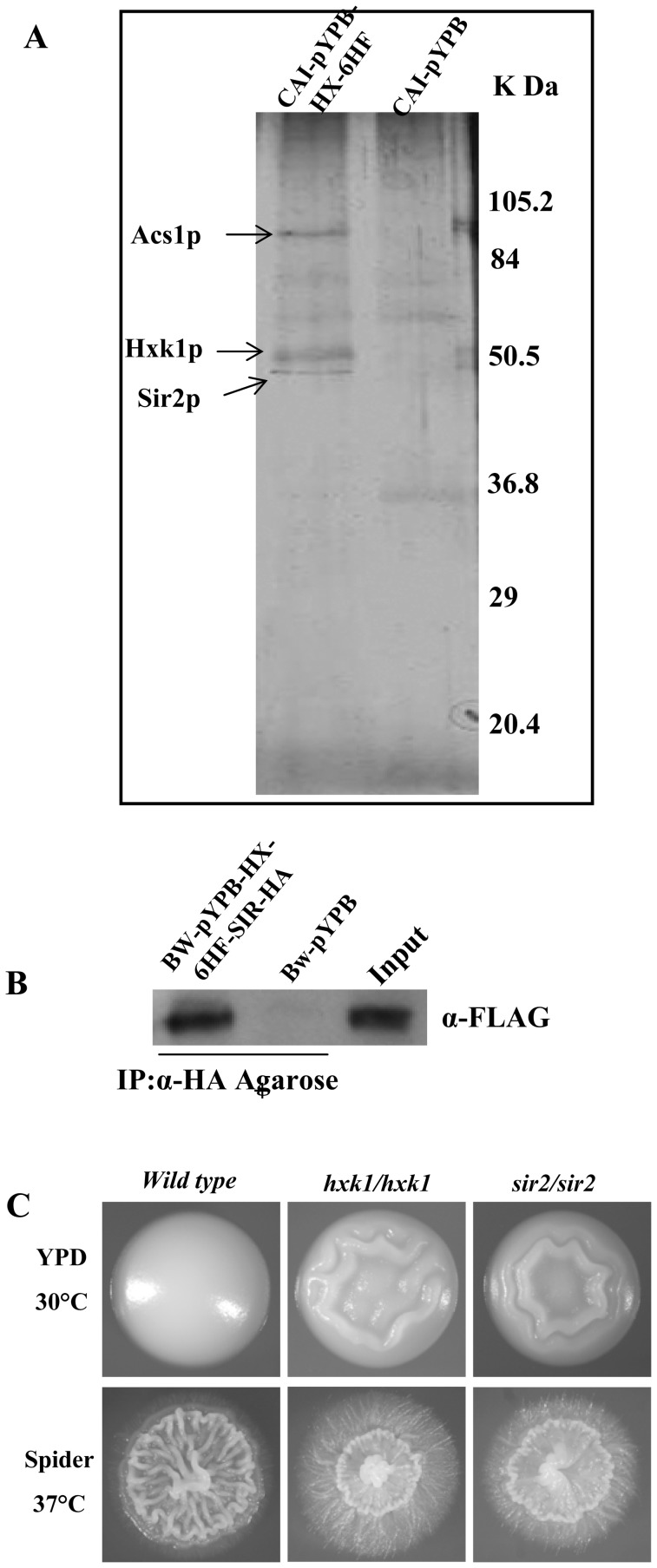
Hxk1p interacts with Histone Deacetylase (Sir2) to repress filament specific genes. A) The Hxk1p complex was purified by tandem affinity purification (TAP-tag) using anti- FLAG and Ni-nitrilotriacetic acid (Ni-NTA) agarose. Protein fractions of purified samples from 1st round with Anti-FLAG Agarose and 2nd round with Ni-NTA Agarose were separated by 12% sodium dodecyl sulfate-polyacrylamide gel electrophoresis and visualized by silver staining. Open arrowheads indicate the component of the Hxk1p complex identified by matrix-assisted laser desorption ionization–time of flight mass spectrometry. CAI-pYPB (control, CAI-4 transformed with pYPB-ADH1-pt, empty vector) and CAI-pYPB-HX-6HF (TAP tagged HXK1expressed under ADH1 p in CAI-4) are Spider medium induced cell extracts. B) Hxk1 interaction with Sir2 confirmed by Co-Immunoprecipitation. BW-pYPB-HX-6HF-SIR-HA and BW-pYPB strain cells were induced in Spider and crude extract isolated as described in Experimental procedures. Immunoprecipitation was carried out in two reactions with anti-HA-Agarose. The crude extract was used as an input. Immunoblotting was performed with anti-FLAG antibody. C) *sir2* mutant showed hyperfilamentation similar to that of *hxk1* mutant in inducing and non-inducing media. Upper panel shows the morphology of wild type, *hxk1* mutant and *sir2* mutant grown on YPD plates at 30°C for 3days and lower panel shows the morphology of wild type, *hxk1* mutant and *sir2* mutant grown on Spider plates at 37°C for 5 days.

To identify a CaHxk1 binding partner that could be involved in the transcriptional repression for the hyphal program, Tandem Affinity Purification (TAP) was performed with cells grown in Spider medium. Crude extracts prepared from a strain in which CaHxk1 was tagged with the 6xHis–FLAG epitope sequence and expressed under ADH1 promoter (details in materials and methods) were subjected to TAP procedures, leading to the detection by SDS-PAGE electrophoresis of proteins composing a CaHxk1p complex (Figure 5). Proteins from the purified Hxk1p complex were identified by peptide mass fingerprinting using matrix-assisted-laser desorption ionization-time of flight mass spectrometry. Proteins approximately 55 kDa and 60 kDa in size turned out to be CaHxk1 and Sir2 histone deacetylase (a member in SIR family histone deacetylase) respectively. Another sharp band having an apparent molecular mass of 80 kDa could be assigned to *C.albicans* Acs1 (Acetyl co-A Synthase). The occurrence of a histone deacetylase as a complex with Hxk1 appeared to be quite significant. Hxk1 interaction with Sir2 was confirmed by Co-Immunoprecipitation (Figure 5 B).

### Hxk1 is Involved in the Cell Wall Synthesis

**Figure 6:**
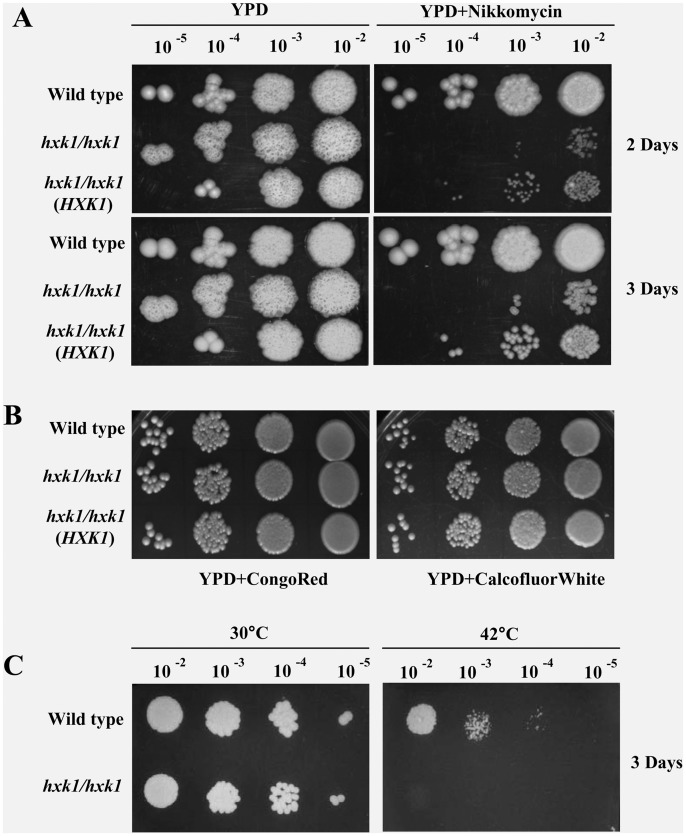
Response of *hxk1* response to cell wall perturbing agent and high temperature. Wild-type strain, *hxk1* mutant and revertant strains were grown in YPD for 8 hr at 30°C and 10-fold dilution series was spotted on YPD, YPD+ Nikkomycin Z (20 µg ml^−1^), YPD_+_ Congo Red (100 µg ml^−1^) and YPD+Calcofluor white (30 µg ml^−1^) plates at indicated concentrations(A and B). For temperature shift experiments, spotted plates were kept at 30°C and 42°C.

Since GlcNAc is a monomeric unit of chitin present in the cell wall of yeast cells, we checked the sensitivity of *hxk1* mutant against cell wall perturbing agents. The mutant showed an increased sensitivity to nikkomycin Z (20 µg ml^−1^), a competitive inhibitor for Chitin synthase, but not to other cell wall perturbing agents like Congo Red (100 µg ml^−1^) and Calcofluor White (30 µg ml^−1^ ) (Figures. 6A and 6B). Reintroduction of the *HXK1* gene at the native locus partially alleviated the sensitivity to Nikkomycin Z. The *hxk1* mutant was also sensitive to a temperature shift of 42°C (Figure 6C).

### Hxk1 Shows Dynamic Changes in Subcellular Distribution

**Figure 7:**
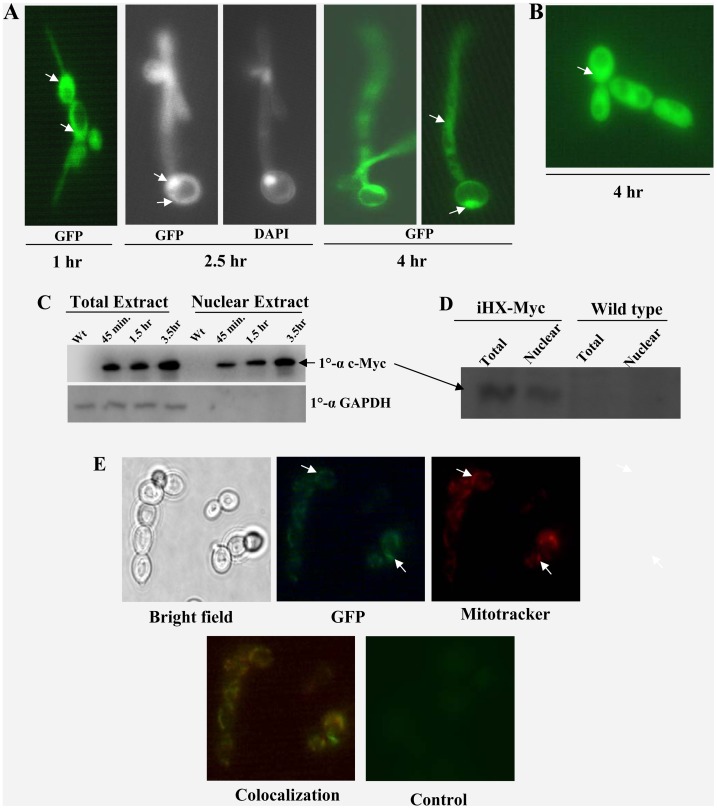
HXK1 localized in the cytoplasm, nucleus and mitochondria in condition specific manner. Sub-cellular localization of Hxk1p-GFP in wild-type cells induced in GlcNAc 2.5 mM or 2% and non-fermentative carbon sources like ethanol or glycerol, 5%. iHXK1-GFP along with wild type (control) grown for 6 h in YPD at 30°C and then, washed in water and induced in GlcNAc 2.5 mM (salt base) or 2%. Cells were visualized either by bright field or by epifluorescence at 1 hr, 2.5 hr and 4 hr in 2.5 mM GlcNAc (A), and 4 hr in 2% GlcNAc (B) after induction. The nuclear localization of Hxk1p was further confirmed by western blotting analysis. iHXK1-Myc strain was induced in 2.5 mM GlcNAc (C) or 2% glucose (D), nuclear and total cellular fractions were checked for presence of hxk1p with help of anti c-myc antibody. The purity of nuclear fraction (free of cytoplasmic contamination) was checked by using Anti-GAPDH antibody specific for cytoplasmic fraction. To check localization in non-fermentable carbon sources, cells were grown for 6 h in YPD at 30°C, washed in water and induced in YNB w/o amino acid with 5% ethanol (E). Hxk1 localization is marked with white arrowheads.

Protein localization data is one of the important information helpful in elucidating eukaryotic protein function. For this purpose, we chromosomally tagged the *HXK1* at the C-terminal region with GFP sequence by following PCR based strategy [29]. C-terminal Hxk1-GFP-fusion protein is localized in cytoplasm and nucleus in 2.5 mM GlcNAc, a salt based filamentation inducing medium (Figure 7A) whereas in 2% GlcNAc, where catabolism is more prominent, a major fraction of this protein is seen to be present in cytoplasm (Figure 7B). However, in glucose, Spider and Serum grown cells, no detectable signals were observed, since expression levels were comparatively less. In order to investigate the regulatory role of *Candida HXK1* in filamentation repression or glucose repression, nuclear and total cellular fractions were checked for the presence of Hxk1 with cells grown in glucose or GlcNAc respectively through western blot analysis. We found that Hxk1 protein was present in both the fractions under above mentioned conditions (Figures 7C and 7D). The purity of the nuclear fraction was confirmed by simultaneously probing the blot with 1°-α GAPDH. Interestingly, Hxk1 protein was localized to mitochondria in non-fermentative carbon sources like ethanol (Figure 7E). This was further confirmed by co-localization studies with mitochondrial marker (Figure 7E). Thus dynamic changes in the subcellular localization of protein suggest its multiple roles in cellular physiology.

### CaHXK1 Acts both as Repressor and an Activator of Genes Involved in Various Cellular Processes Apart from Regulating GlcNAc Metabolic Genes

Apart from their catalytic function, metabolic enzymes have acquired important regulatory functions controlling metabolism, stress resistance, growth and development in bacteria, yeasts, plants and animals [30], [31], [32]. To elucidate the role of GlcNAc kinase in sugar signaling in *Candida albicans*, we used microarray for expression profile study.

**Figure 8:**
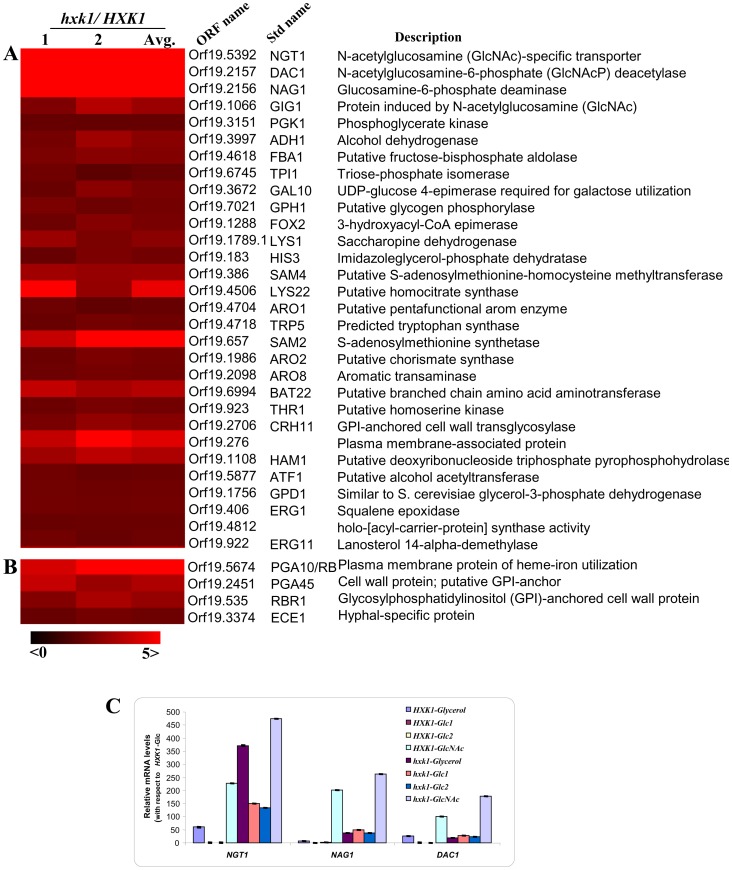
Transcriptome analysis of *HXK1* regulated genes. A) Partial Heat map of over-represented genes (p-values <0.05, False Discovery Rate <1% in GO term analysis) differentially expressed in a *HXK1* dependent manner in response to glucose. Two-color microarray data expressed as *hxk1*/*HXK1* ratio (1 and 2 are biological replicates with dye swap, average of 1 and 2, *Ave*) is plotted as heat map. The color scale at the bottom indicates the log2 ratio. B) Heat map of Hyphal specific genes (HSGs) differentially expressed in a *HXK1* dependent manner in response to glucose. C) Modulation of expression levels of GlcNAc catabolic genes by *HXK1.* Quantitative RT-PCR of GlcNAc catabolic gene transcripts *NGT1*, *NAG1* and *DAC1* in *C. albicans* wild type (CAF2–1) and *hxk1* mutant, cells in response to glycerol-6%, Glucose-2%, Glucose-5 mM or GlcNAc-5 mM. *ACT1* has been selected as the endogenous control. The error bars represent co-efficient of variation.

A comparative transcriptome analysis carried out with wild type and *hxk1* mutant cells grown in 2% glucose (YNB-Glucose) at 30°C revealed differential regulation of several genes (Figure 8A and Table S2) involved in various cellular processes. GO term analysis (CGD site) helped us to functionally categorize these genes (Table S2) into over-represented groups viz., carbohydrate metabolic process (GlcNAc metabolism and glycolysis), organic acid metabolic process, amino-acid metabolic process etc. Hyphal specific genes (HSGs) (Figure 8B) were also present in the differentially upregulated genes even though these genes did not fall in the over-represented functional category (GO term analysis). The down regulated genes in the array data are mostly uncharacterized and could not be classified in to any functional category and hence omitted from the GO term analysis. Since in this array data-set, an important category comprised of the GlcNAc metabolic genes (Figure 8A and Table S3), we were interested to see the other Hxk1 regulated genes’ correlation with the GlcNAc mediated response. A transcriptome profile analysis between GlcNAc vs. glucose and GlcNAc vs. Glycerol (glycerol was selected keeping in mind the strong catabolite repressor effect of glucose) grown cells for wild type helped us to have an overview of role of Hxk1 in GlcNAc metabolism (Figure S1).

To gain further insights into *HXK1* mediated GlcNAc catabolic gene expression we performed q-RT PCR analysis of the genes like *NGT1, NAG1* and *DAC1* in presence of 5 mM glucose or GlcNAc that mimicked physiological conditions. Simultaneously to nullify catabolic repression we grew the wild type and mutant cells in glycerol and induced in glucose 2% or 5 mM Glucose or GlcNAc 5 mM. In *hxk1* mutant the levels of derepression (*NGT1–* 140; *NAG1*–50; *DAC1*–30 folds) of these GlcNAc catabolic genes were comparable to the fold of induction (*NGT1–*220; *NAG1*–200; *DAC1*–100 folds) in the wild type GlcNAc grown cells (Figure 8C). While such a kind of result shows the Hxk1 dependence of these genes, it also brings into picture some additional means of regulation of their expression. This result also reveals another interesting fact that Hxk1 participates in maintaining the optimum levels of gene expression by exerting repressive effects on GlcNAc catabolic genes in presence of this amino sugar.

## Discussion

### Hxk1 Orchestrates Morphogenesis

The response of organisms to environmental cues is often determined by the orchestration of different transcriptional circuits. We provide several lines of evidence to show that *HXK1* mediated hyperfilamentation is quite unique and independent of the classical regulators. Moreover, an upregulation in the levels of *HXK1* during filamentation induction probably hints at it its role in maintaining an optimum level of expression of the filament specific genes through their repression.

Recent studies indicate that morphological transitions are controlled at chromatin level through regulation of histone acetylases and methyltransferases [33], [34]. At this point our finding of Hxk1 interaction with Sir2 could be quite significant. In *C. albicans SIR2* has already been reported to take part in heritable changes in the developmental pathways involved in the bud–pseudohyphae–hyphae transitions [35] and white-opaque switching. Thus, Hxk1 by interacting with Sir2 probably modulates its activity at chromatin level to repress the HSGs expression although such a conclusion is quite preliminary. Furthermore, an overall similarity in the morphology of *sir2* and *hxk1* mutant under both non-inducing (YPD 30°C) and inducing (Spider 37°C) indicated a probable involvement of Sir2 mediated repression of Hxk1 dependent filamentation (Figure 5C). Physiologically, this Hxk1 mediated filamentation repression could be important for maintaining balanced state during filamentation conditions.

### Hxk1 has Multifarious Roles

**Figure 9:**
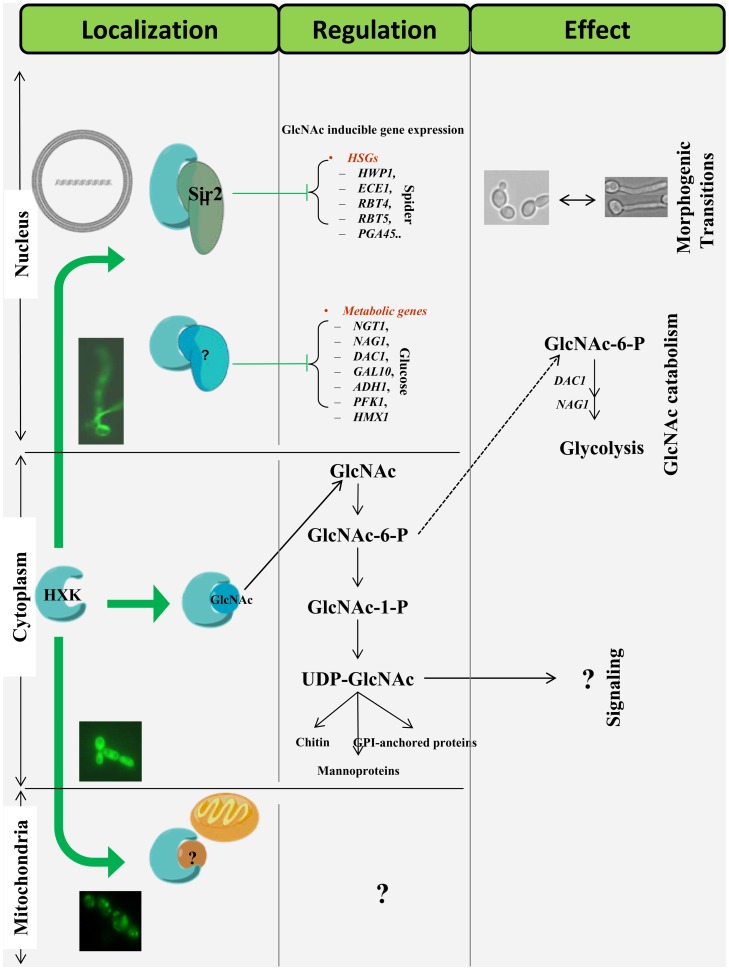
A brief overview of mode of action, localization, targets and downstream effects of Hxk1. The protein capable of multifarious localization migrates to nucleus where it possibly interacts with a Histone Deacetylase Sir2 to keep the HSGs in a repressed state. It probably partners again with Sir2 or some other proteins to repress the GlcNAc metabolic genes while still in nucleus. In cytoplasm Hxk1 phosphorylates GlcNAc to carry out the catabolism of this amino sugar and contributing probably to the overall UDP-GlcNAc pool within the cell. In presence of non-fermentable carbon sources like ethanol or glycerol the protein migrates to mitochondria where its mode of functioning is still not known.

The dynamic localization of this particular protein under different conditions also led us to presume its contribution to controlling various phenomena within the cell. A cytoplasm localized Hxk1 in presence of GlcNAc or Glucose could be effectively participating in GlcNAc catabolism and additionally maintaining the UDP-GlcNAc pool within the cell. While in nucleus in response to spider, Glucose or GlcNAc, the protein might be playing a role in maintaining the filamentation specific genes or GlcNAc catabolic genes in a repressed state. A change in the carbon source to a non-fermentable one, initiated a mitochondrial localization, presumably due to the involvement of Hxk1 in cellular respiration. Such functional plasticity of the protein in keeping with its localization to more than one cellular compartment has been outlined in Figure 9.

In a separate study it was indicated that disruption of *HXK1* resulted in severe attenuation of virulence when compared with *NAG1* or *DAC1* disruptions [36]. The severity of attenuated virulence in *hxk1* mutant might be correlated to *HXK1*’s role in GlcNAc catabolism and anabolism [37], [38] apart from its regulation of expression of various cell wall proteins or virulence factors, iron responsive genes, mitochondrial genes, amino-acid biosynthesis and many uncharacterized genes as seen in our microarray data (Figure 8). The cell wall perturbing agent Nikkomycin Z inhibits the biosynthesis of chitin in cell walls due to its structural resemblance to UDP-N-acetylglucosamine. An hxk1 mutant showed increased sensitivity to Nikkomycin Z due to an overall decrease in the pool of UDP-GlcNAc within the cell via the Hxk1 mediated phosphorylation of GlcNAc. GlcNAc 6–phosphate can enter a catabolic route that links hexosamine metabolism with the glycolytic pathway [39] or may enter an anabolic pathway leading to UDP-GlcNAc formation [40].

Overall, in addition to its well established role in metabolism we report *HXK1* to have significant role in transcriptional regulation, and maintaining important cellular functions. The multifarious roles of Hxk1, achieved through its dynamic subcellular localization and regulation of genes involved in various cellular processes especially metabolic gene regulation could be an adaptive advantage for this human fungal pathogen. It is also plausible that such a direct level of regulation might confer a selective advantage in terms of swiftness and flexibility in the response.

### Hxk1 and GlcNAc Signalling

This enzyme is at the gateway to GlcNAc signaling. We propose a model for the mode of action of *HXK1* (Figure S2), by taking into account our observations and available literature [41], [42], [43]. In the absence of glucose or other sugars, or presence of GlcNAc, *NGT1* is relieved from Hxk1 repression (Figure 8B); GlcNAc enters the cell and induces GlcNAc catabolic/metabolic genes. *HXK1* phosphorylates GlcNAc and probably induces *NAG1* and *DAC1* genes. In addition to *HXK1* mediated coordination, freely entered GlcNAc is also able to induce gene expression as described in the recent literature by Naseem *et al* (2011). But, the GlcNAc sensor yet remains to be identified. None of the classical regulators (*HGT4, RGT1, MIG1,* and *TUP1*) seem to play role in the regulation of these catabolic genes (data not shown). There could be the probable involvement of uncharacterized regulator/s. (GlcNAc also might directly interact with inducers to modulate the GlcNAc catabolic gene expression). Thus it seems, that GlcNAc as a molecule evokes some novel responses, the signaling mechanism of which is not yet fully understood.

Thus, fine tuning of cellular functions emerges to be important for utilizing the resources in economic way and to orchestrate various functions in a co-ordinated fashion. An understanding of the regulatory aspects of metabolic enzymes like hexokinase in broader perspective is needed to elucidate the molecular mechanism/s involved in sugar sensing pathways in yeasts and mammals.

## Materials and Methods

### Media and Growth Conditions

Standard media YPD and SD [44] were used routinely for strain growth and maintenance. Selections for *URA3* prototrophy and counter selection against -*URA3* were performed as described earlier [45]. Spider-37°C [44], 2.5 mM GlcNAc in salt base, containing 0.45% NaCl, and 0.335% YNB w/o amino acids- 37°C [2], YPD with 20% bovine serum-37°C or corn meal agar-Tween-80 (1%) [46] were used for the colony growth assays and also for filamentation induction. For liquid morphological study and northern analysis, YPD (30°C) was used to pre grow cells to saturation overnight. 1% pre-culture was added to YPD as bulk culture, and allowed to grow 5–6 hrs till it reached exponential growth phase. Then, cells were harvested by centrifuging at 6,000 rpm for 5 minutes, and were washed twice with sterile MQ-water. They were resuspended in 5ml sterile MQ-water and starved for 10 hours at 30°C, with shaking at 120 rpm [47]. The starved cells were resuspended at the concentration of 0.5OD_600_ ml^-1^ in various induction media. They were induced at 37°C for 4 hours in case of GlcNAc, for 2 hours at 37°C in case of Spider and Serum and 2 hours at 30°C in case of YPD.

For colony growth morphological study, YPD, Spider, SLAD and minimal SD plates were used for the colony growth assays. SLAD plates at a concentration of 40 to 50 cells per plate, and incubated at 37°C for 10 days. For induction on Spider medium plates, the cells were grown in Spider medium for 5 days at 30°C, counted, plated at a concentration of 40 to 50 cells per plate, and incubated at 37°C for 7 days [6], [7], [11].

Sensitivity to Nikkomycin Z, Congo Red and Calcofluor White was tested by spotting dilutions of cells onto YPD plates prepared with the indicated concentration of the corresponding chemical. Three microliters of a 10-fold dilution series of cells was spotted on the surfaces of the plates. The plates were then incubated at 30°C for the indicated time and photographed. In case of high temperature (42°C) sensitivity assay, plates were incubated for 2–4 days at 30°C and 42°C.

### Strains and Plasmids

We followed the technique of Fonzi and Irwin to delete genes in *C.albicans*. Both *CaHXK1* alleles in the strain *CAI4* were disrupted and confirmed by Southern blotting analysis [48], [49]. Revertant for sole *hxk1* mutant and *hxk1* double mutants under the background of filamentation pathway specific mutants were created by reintroduction of a functional copy of *HXK1* at the native locus. The details of the disruption and reintegration have been included in the supplemental section. The s*ir2* mutant was also prepared under *CAI4* background (details are given in supplemental section). The strains and plasmids used have been mentioned in Table S4.

### Epitope Tagging

For GFP tagging we used the plasmid pGFP-URA3, which was a kind gift from Cheryl A. Gale (Maryam *et al*., 2001). This plasmid harbours a codon optimized GFP sequence along with *URA3* marker and a *C.albicans ADH1* terminator after the GFP sequence. PCR was performed using this cassette as template and appropriate gene specific primers namely HX-GP-F1 and HX-UR-R1. The PCR product was used for transforming a *C.albicans ura* strain, *CAI4* (Table S4). Transformants were selected by plating the transformation mix on the appropriate selective medium. To identify transformants in which the cassette had correctly integrated into the target gene sequence, genomic DNA was prepared and used as the template in PCR reactions, using one primer that annealed within the transformation module (Tag1) and a second primer that annealed to the target gene locus outside the altered region (Tag2). The same strain (iHXK1-GFP) was reconfirmed by Western analysis using anti-GFP antibody.

To tandemly tag CaHxk1p with the HF (6xHis and Flag) epitope in the genomic locus, a DNA fragment containing the 3′ region of *HXK1*, the 6xHF tag sequence, the *ACT1* terminator, the *URA3* marker, and the downstream region of *HXK1*was amplified, using p6HF-ACT1 as a template (a gift from Prof. Masakazu Niimi), and HX-HFL-F, HX-HFL-R as primers and introduced into *CAI4* to generate iHXK1-HF in an analogous way as described above. Similar protocol was followed to create myc tagged *HXK1* strain (iHXK1-Myc) by using pFA5a-13Myc-URA3 (primers used: HF-F2 and HX-UR-R1) as template.

To express TAP tagged HXK1 protein under ADH1 promoter in *Candida albicans,* pYPB1-ADHpt-HXK1-HF plasmid was transformed into CAI4. To construct plasmid pYPB1-ADHpt-HXK1-HF carrying TAP tagged *HXK1* under control of the *ADH1* promoter, the coding region of *HXK*1 including C-terminally fused TAP tag (6xHis-FLAG) was amplified from iHXK1-HF (using primers HX.AD.HF1 and HX.AD.R) and cloned into the *Bgl*II site of the *C. albicans* expression vector pYPB1-ADH pt as described elsewhere [50].

For Co-IP, BW-pYPB-HX-6HF-SIR-3HA strain in which SIR2 was tagged with 3xHA sequence and pYPB-HX-6HF plasmid was co-transformed in *BWP17*. To create HA tagged *HXK1* strain (iHXK1–3xHA) pFA5a-3xHA–URA3 plasmid was used (primers used: HF-F2 and HX-UR-R1) as template. In control strain BW-pYPB, empty plasmid pYPB was transformed in *BWP17*.

Sequence of all the primers mentioned in the text have been included in Table S5.

### Western Blot Analysis and Immunoprecipitation

Cell pellets induced in Spider medium were collected and total crude extracts were prepared by vortexing with glass-beads. Western blot analysis was carried out using 15 µg of protein from each sample to assess protein levels of Hxk1 at indicated time points. The blots were probed with 1°α-c-myc.

For Western blot analysis, proteins from gels were electrotransferred to Hybond C Extra membrane (Amersham Biosciences). The blot was blocked using 3% skimmed milk in a buffer containing 10 mM Tris, pH 7.4, 15 mM NaCl, 0.05% Tween-20. For detection of proteins tagged with c-myc epitope, membranes were incubated with anti-c-myc monoclonal antibody (Sigma), at a dilution of 1∶1000 and a peroxidase conjugated secondary antibody (Amersham Biosciences, UK) at a dilution of 1∶20000. An enhanced chemiluminescence (ECL+) detection system (Amersham Biosciences, UK) was used for antibody detection according to the manufacturer’s instructions.

Two-step purification was done as described by Kaneko *et al.* [51] with little modifications. Cells induced in spider were harvested by centrifugation, washed with NP-40 buffer, and frozen on liquid nitrogen. The frozen cells, resuspended in NP-40 buffer (50 mM NaH_2_PO_4_, pH 8.0, 150 mM NaCl, 1% Nonidet P- 40) containing a protease inhibitor cocktail (complete, EDTA-free tablet, Roche Diagnostics, Germany) and PMSF-protector solution (PMSF PLUS, Roche Diagnostics), were lysed at 4°C by using bead beater for 10 cycles of 30 sec burst and 120 sec gap and then centrifuged at 12000 rpm to remove insoluble material. Anti-FLAG M2 affinity agarose (Sigma) or Ni-NTA agarose (Qiagen) was washed and equilibrated three times with the NP-40 buffer prior to use. The cell lysates were first subjected to anti-FLAG M2affinity agarose and the eluate was applied to Ni-NTA agarose. 10–12 mg of protein was incubated with 50 µl preequilibrated anti-Flag M2 affinity beads (Sigma-Aldrich) overnight at 4°C. The beads were then washed 4 times using 10 bed volumes of NP-40buffer and proteins were eluted using 100 µl of NP40 buffer containing 400 µg/ml of FLAG peptide (Sigma-Aldrich). The eluted proteins were then bound to 20 µl pre-equilibrated Ni-NTA beads (Qiagen) for 2 hrs at 4°C. After 4 washes with 10 bed volumes of NP40 buffer, proteins were eluted in two aliquots of 50 µl of 300 mM imidazole in NP40 buffer. Residual agarose beads were removed by passage of the eluate through Ultrafree-MC centrifugal Filter Units (Millipore, MA, USA). Five times sample buffer (2% SDS, 10%glycerol, 40%mMTris pH 6.8, 715 mM β-mercaptoethanol) was added to the eluted material and heated for 10 min at 65°C prior to SDS-PAGE. The lane containing the resolved proteins was excised from the gel and sliced horizontally into gel slices of ∼1.5 mm and subjected to trypsinolysis according to standard techniques [52]. The tryptic peptides were extracted and loaded onto C18 PepMap 100, 3 µm (LC Packings), and separated with a linear gradient of water/ACN/0.1% (v/v) formic acid. The peptides were analyzed using a QSTAR Elite mass spectrophotometer (Applied Biosystem) coupled with an online Tempo nano-MDLC system. The acquired mass spectra were searched against the fungal database using Mascot search engine27 (www.matrixscience.com). The database search parameters were: taxonomy set to Fungi; peptide tolerance, 100 ppm; fragment mass tolerance, 0.4 Da; maximum allowed missed cleavage 1; fixed amino acid modification as carbamidomethyl and variable amino acid modifications either oxidation (M) or acetyl (N-term), or both. Protein scores were derived from ion scores as a nonprobabilistic basis for ranking protein hits. Proteins were assigned as identified if the MOWSE score27 was above the significance level provided by the Mascot search algorithm.

For co-immunoprecipitation, BW-pYPB-HX-6HF-SIR-3 HA and BW-pYPB strains cells were induced in Spider, harvested by centrifugation, washed with NP-40 buffer, and frozen in liquid nitrogen. The frozen cells, resuspended in NP-40 buffer (50 mM NaH_2_PO_4_, pH 8.0, 150 mM NaCl, 1% Nonidet P- 40) containing a protease inhibitor cocktail (complete EDTA-free tablet, Roche Diagnostics, Germany) and PMSF-protector solution (PMSF PLUS, Roche Diagnostics), were lysed at 4°C by using bead beater for 10cycles of 30sec burst and 120 sec gap and then centrifuged at 12000 rpm to remove insoluble material. 10 mg crude lysate was incubated with 50 µl anti-HA Agarose (Roche) for 2 hrs at 4°C. The beads were washed, boiled resolved on 11% SDS-PAGE gel. Hxk1was detected by Western blot analysis with anti-FLAG antibody. Immunoprecipitation experiments were performed in duplicates.

### RNA Expression Analysis

By using appropriate primers pairs (sequences are provided in Supplemental Section) Probe DNA’s were amplified from SC5314 genomic DNA, the resulting product purified using a QIA quick column according to manufacturer’s instructions, and a^ 32 ^P probe made using a random priming kit from NEB. *C.albicans* cells were grown as specified above and whole RNA extracted using hot phenol method. Briefly, 20 µg total RNA was loaded on a 1.5% formamide- agarose gel, transferred onto a Hybond-N+ nylon membrane by capillary blotting [53], and fixed by UV cross-linking. Identical aliquots were run on each gel; blots were used not more than twice, prehybridizing and hybridizing at 42°C, and washing at same temperature. Autoradiograms were digitized using an alpha Imager scanner and their backgrounds were adjusted in Photoshop.

### Subcellular Fractionation

Cells were harvested and treated to obtain sphaeroplasts with Lyticase (Sigma) in Zymolyase buffer (50 mM Tris HCl [pH 7.5], 10 mM MgCl_2,_ 1 M sorbitol, 1 mM dithiothreitol) at 30°C for 4 hr with mild shaking. The sphaeroplast suspension was introduced drop by drop into a beaker containing Ficoll buffer (18% [wt/vol] Ficoll-400, 10 mM Tris HCl [pH 7.5], 20 mM KCl, 5 mM MgCl_2_, 3 mM dithiothreitol, 1 mM EDTA) containing 1 mM PMSF. The diluted solution was centrifuged at 20,000 *g* for 20 min at 4°C, and the supernatants were used for Western analysis as a cytoplasmic fraction. The resultant pellets were resuspended in Ficoll buffer and used for Western analysis as a nuclear fraction.

### Microscopy

Microscopic images of yeast cells were captured using a Nikon 80 i inverted microscope equipped with a Nikon Digital DXM1200C camera and Stereo microscope (Nikon SMZ 1500) using ×40, ×100 or ×7 objective and differential interference contrast optics when required. The Nikon 80 i photomicroscope was equipped with a 100 W mercury lamp, and epifluorescence illumination with green fluorescent protein (GFP) (excitation filter 470–490 nm, barrier 520–580 nm) blue fluorescent protein/cyan fluorescent protein (CFP) (excitation filter 380–400 nm, barrier 435–485 nm) and yellow fluorescent protein (YFP) (excitation filter 490–510 nm, barrier 520–550 nm) filter sets. For co-localization studies Mito-Tracker Red CMX Ros (Molecular Probes, Invitrogen) was used according to manufacturer’s instructions and cells were stained with this dye for 10 mins under proper growth conditions (YNB w/o amino acid with 5% ethanol at 30°C). Digital images were collected using a Cool Cam liquid-cooled, three chip colour CCD camera (Cool Camera Company, Decatur, GA) and captured to a Pentium II 300 MHz computer, using Image Pro Plus version 4.1 software (ACT1). Images were processed using Adobe Photoshop version 7.0 (Adobe Systems Corp., San Jose, CA).

### RNA Extraction and RT-PCR Analysis

*C. albicans* strains were grown as described under “Immunoblotting section in a YNB- basal medium containing 6% glycerol and induced in Glycerol (6%), glucose (2% or 5 mM) and GlcNAc (5 mM). Cells were harvested rapidly by filtration and snap frozen in liquid nitrogen vapours. Total RNA was isolated using hot phenol method and the concentration was determined using Nanodrop spectrophotometer. For all RT-PCR experiments, total RNA was treated with RNase-free DNase I (Invitrogen) to remove any residual DNA. About 500 ng of DNase I-treated RNA was used for single-stranded cDNA synthesis in 10 µl of reaction mixture using a High-Capacity cDNA Reverse Transcription kit (Applied Biosystems) and used for qRT-PCR with SYBR green PCR master mix on an ABI Prism 7000 real-time PCR apparatus (Applied Biosystems). The comparative *CT* method (2^−ΔΔCT^) was used to determine the relative gene expression [54]. Control reactions without reverse transcriptase were carried out for each cDNA preparation and ascertained that no amplification was obtained as judged by high *CT* [55] values and gel analysis.

### Microarray Experiment

The DNA oligonucleotide microarray was procured from Genome Sequencing Centre at Washington University, St.Louis, USA. In the array each ORF is represented by a specific 70-mer oligonucleotide, and one genome equivalent was spotted three times per slide (i.e., n = 3 for each hybridization) [43].

For induction, *Candida albicans* wild type, SC5314 and *hxk1* mutant, H8–1–103, were grown in YNB (w/o aa:0.67%) plus 2% glucose at 30°C in a shaking incubator to mid-log phase. The cell pellets were washed once with YNB (w/o aa: 0.67%) and were put in YNB (w/o aa: 0.67%) plus 2% glucose media. At 30 min time point cells (≈20 OD) were collected rapidly by filtration and snap frozen using liquid nitrogen vapors and stored in −80°C till used.

Total RNA was isolated using the hot SDS-phenol method as described below. Frozen cell pellets (OD≈30) were suspended in 12 ml of AE buffer, pH5.0 (for 500 ml Buffer: 8.330 ml Sodium acetate-3 M, pH5.3+10 ml EDTA-0.5 M, pH8.0) at room temperature, after which 1 ml of 20% sodium dodecyl sulfate and 12 ml of acid phenol (Fisher Scientific, Waltham, MA) was added. This mixture was incubated 15 min. at 65°C with vortexing after each 5 minute, cooling on isopropanol slush for 2–3 min, and finally centrifuging for 15 min at 10,000 rpm, 20°C. Supernatants were transferred to new tubes containing 15 ml of chloroform, mixed and centrifuged at 1500 rpm for 10 min, 20°C. The aqueous layer was removed to new tubes, RNA precipitated with 1 volume isopropanol and 0.07 vol. of 3 M sodium acetate (pH5.3), and then collected by centrifugation at 12,000 rpm for 35 min at 4°C. The RNA pellet was suspended in 10 ml of 70% ethanol, collected again by centrifugation, dried and suspended in formamide or nuclease free water. RNA purification and quantification-The RNeasy Mini Kit (#74104) was used as per instructions of manufacturers to clean up RNA isolated by hot phenol method. Checked RNA concentration and quality by measuring OD_260_ and OD_260/280_ of 1.5 µl sample using NanoDrop spectrophotometer.

The first strand cDNA synthesis, second-strand cDNA synthesis, *in-vitro* transcription to synthesize amino-allyl modified aRNA and subsequently amino-allyl modified aRNA –dye coupling was done using Ambion MessageAmpII Amino Allyl kit with Cy Dyes (#1753).

The microarray slides were prehybridized in 5× SSC, 0.1 mg/ml BSA and 0.1% SDS at 42°C for 1 h. Slides were washed 3–4 times in sterile fresh water at RT and dried by centrifugation at 200 g for 5–10 min in a 50 ml falcon tube layered with Kim wipe at its base as cushion. Prior to hybridization, ≈125 pico moles each (dye content) of Cy5 and Cy3 labeled aRNA samples were combined and aRNA fragmented in 10 µl using RNA fragmentation Kit (Ambion) at 70°C for 15 min according to manufacture’s instructions. The samples were placed on ice until ready to use or stored at −80°C. The fragmented aRNA was denatured at 65°C for 5 min. and hybridized to *C.albicans* microarray slide in GeneTAC hybridization station (Genomic Solutions) at 42°C for 14–16 hrs. After the hybridization, the slides were washed in a dark staining tray as follows. Wash buffer 1∶2× SSC, 0.2% SDS; Wash buffer 2∶2× SSC; Wash buffer 3∶0.2×SSC. The slides were dried as described earlier.

Scanning and quantification of the hybridized microarray slides were performed using the ScanArray Express software from Perkin Elmer. The microarray slide was scanned at 10 µm with fixed laser power and high and low PMT settings for both Cy5 and Cy3 channels. The ScanArray software was used for gridding and image quantification. The features such as those with artefacts like dust, streaks and odd morphology such as comets were flagged as bad spots. Low intensity and saturated spots were eliminated by flagging spots with spots with signal to noise ratio below 3.0. The Cy5 and Cy3 channels were normalized based on intensity dependent normalization (Loess) in a plot of log (ratio) vs log (mean intensity). Log_2_ ratio of the Cy5/Cy3 intensity for each spot was calculated. The data for triplicate spots (with log2 ratio <1 S.D.) for each gene in each array was merged and used for further statistical analysis. Significance analysis of microarrays [56] was performed as described previously [57], with the exception of 1000 permutations to get differentially expressed genes with low false discovery rate. Heat map of the differentially expressed genes was constructed using MeV v4.8.1 software. The experiment was repeated with freshly labelled RNA from biological replicate and hybridized using the different dye for each biological replicate.

### GO Term Analysis

GO term analysis was performed at CGD (Candida Genome Database) site for differentially upregulated genes with p- value <0.05, False Discovery Rate (FDR) <1% and “Process” was chosen under ontology. The functional categories that are truly over-represented are shown in the table S3.

## Supporting Information

Figure S1 Transcriptome analysis of *hxk1* mutant Ai**)** Partial Heat map showing a comparative profile of *HXK1* mediated few GlcNAc inducible genes. In the comparison between three microarray data sets viz. a) *hxk1* mut vs. wild type in glucose b) GlcNAc vs. glucose for wild type, and c) GlcNAc vs. Glycerol for wild type we found some unique upregulated genes (*NGT1, NAG1, DAC1, GIG1, GAL10, Tca5c, GAP3, ORF19.4792, HSR1, PEX25)* as GlcNAc inducible.(fold change is shown). Aii) Numerical representation of total independent and overlapping sub-sets of upregulated (>2 fold) genes between *hxk1*/*HXK1* and GlcNAc vs. Glucose. B) Heat map showing a comparative profile of few genes differentially regulated between GlcNAc vs.Glucose and GlcNAc vs. Glycerol. Genes like *HGT12, HGT2, HXT5* and *HGT1* that has been reported as GlcNAc induced genes in the study by Gunasekera *et al*.(2010) may not be truly inducible genes. In fact, these genes (except *HGT1*) showed down regulation in GlcNAc when compared with glycerol grown cells. Several other novel genes showed up-regulation in the expression profile when compared between GlcNAc and glycerol (data not shown).
(TIF)

Figure S2 Mode of action of *HXK1.* Hxk1 plays a key role in the GlcNAc entry and GlcNAc induced gene expression. In the absence of glucose or other sugars, or presence of GlcNAc, *NGT1* is relieved from Hxk1 repression (Fig. 9B); GlcNAc enters the cell and induces GlcNAc catabolic genes. In addition, freely entered GlcNAc is also able to induce gene expression as described in the recent literature by Naseem *et al* (2011). But, the GlcNAc sensor yet remains to be identified. Hxk1 also repress GAL10, GIG1, ADH1, PFK1 etc., There could be the probable involvement of uncharacterized regulator/s (GlcNAc also might directly interact with inducers to modulate the GlcNAc catabolic gene expression).
(TIF)

Figure S3 Calcofluor staining of *hxk1* single and double mutants along with wild type. Cells were induced in Spider medium at 37°C for 2 hours and stained with Calcofluor white, which stains chitin in the cell walls and septa. White arrowheads show the septations.
(TIF)

Table S1 Quantitative estimation of filamentation in wild type, *hxk1* single and double mutants.
(DOC)

Table S2 List of significantly upregulated/downregulated genes in hxk1mutant compared to wild type in glucose grown cells.
(XLS)

Table S3 Functional categorization of significantly over represented gens.
(XLS)

Table S4 Strains used in this study.
(DOC)

Table S5 List of primers used in the study.
(DOC)

Text S1 (DOC)
