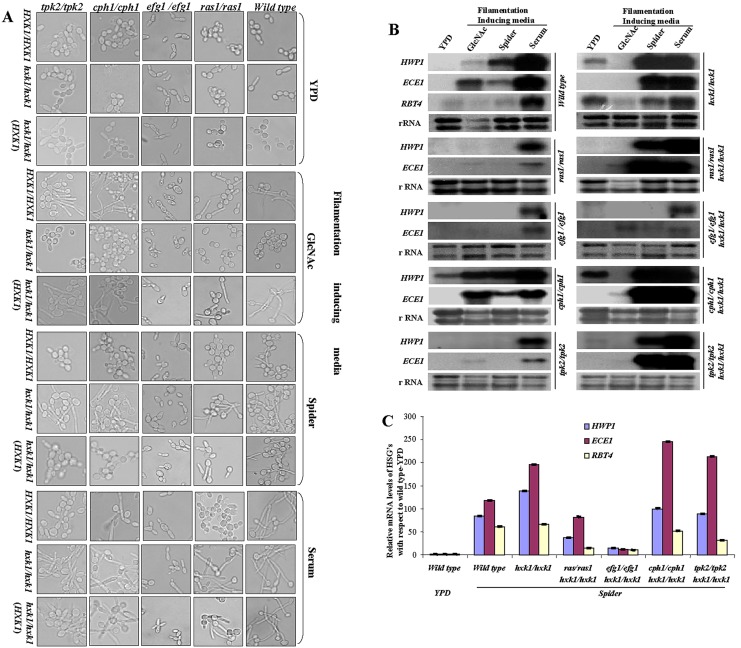# Correction: N-Acetylglucosamine Kinase, *HXK1* Is Involved in Morphogenetic Transition and Metabolic Gene Expression in *Candida albicans*

**DOI:** 10.1371/annotation/c381f4a0-efa6-41e5-8bb5-172c714510b5

**Published:** 2013-10-10

**Authors:** Kongara Hanumantha Rao, Swagata Ghosh, Krishnamurthy Natarajan, Asis Datta

Panels 3B and 3C are missing from Figure 3. Please see the corrected Figure 3 here: 

**Figure:**