# Supplementary figures and images for: N-Acetylglucosamine Kinase, HXK1 Is Involved in Morphogenetic Transition and Metabolic Gene Expression in Candida albicans
Source: PLoS One. 2013 Jan 14;8(1):e53638. doi: 10.1371/journal.pone.0053638 (PMC3544915; doi:10.1371/journal.pone.0053638)

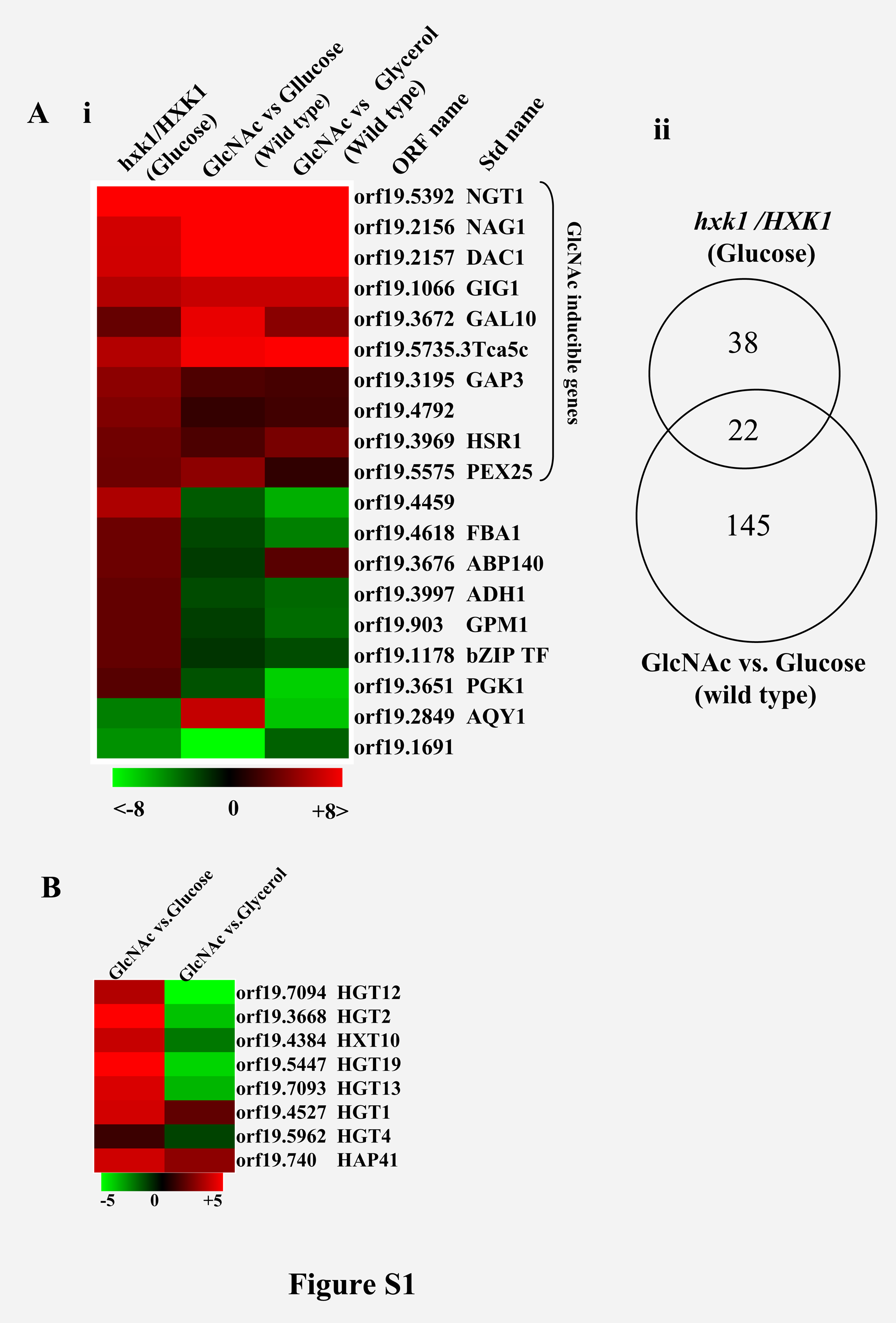

Supplement: Figure S1 — Transcriptome analysis of hxk1 mutant Ai) Partial Heat map showing a comparative profile of HXK1 mediated few GlcNAc inducible genes. In the comparison between three microarray data sets viz. a) hxk1 mut vs. wild type in glucose b) GlcNAc vs. glucose for wild type, and c) GlcNAc vs. Glycerol for wild type we found some unique upregulated genes (NGT1, NAG1, DAC1, GIG1, GAL10, Tca5c, GAP3, ORF19.4792, HSR1, PEX25) as GlcNAc inducible.(fold change is shown). Aii) Numerical representation of total independent and overlapping sub-sets of upregulated (>2 fold) genes between hxk1/HXK1 and GlcNAc vs. Glucose. B) Heat map showing a comparative profile of few genes differentially regulated between GlcNAc vs.Glucose and GlcNAc vs. Glycerol. Genes like HGT12, HGT2, HXT5 and HGT1 that has been reported as GlcNAc induced genes in the study by Gunasekera et al.(2010) may not be truly inducible genes. In fact, these genes (except HGT1) showed down regulation in GlcNAc when compared with glycerol grown cells. Several other novel genes showed up-regulation in the expression profile when compared between GlcNAc and glycerol (data not shown). (TIF) [file pone.0053638.s001.tif]

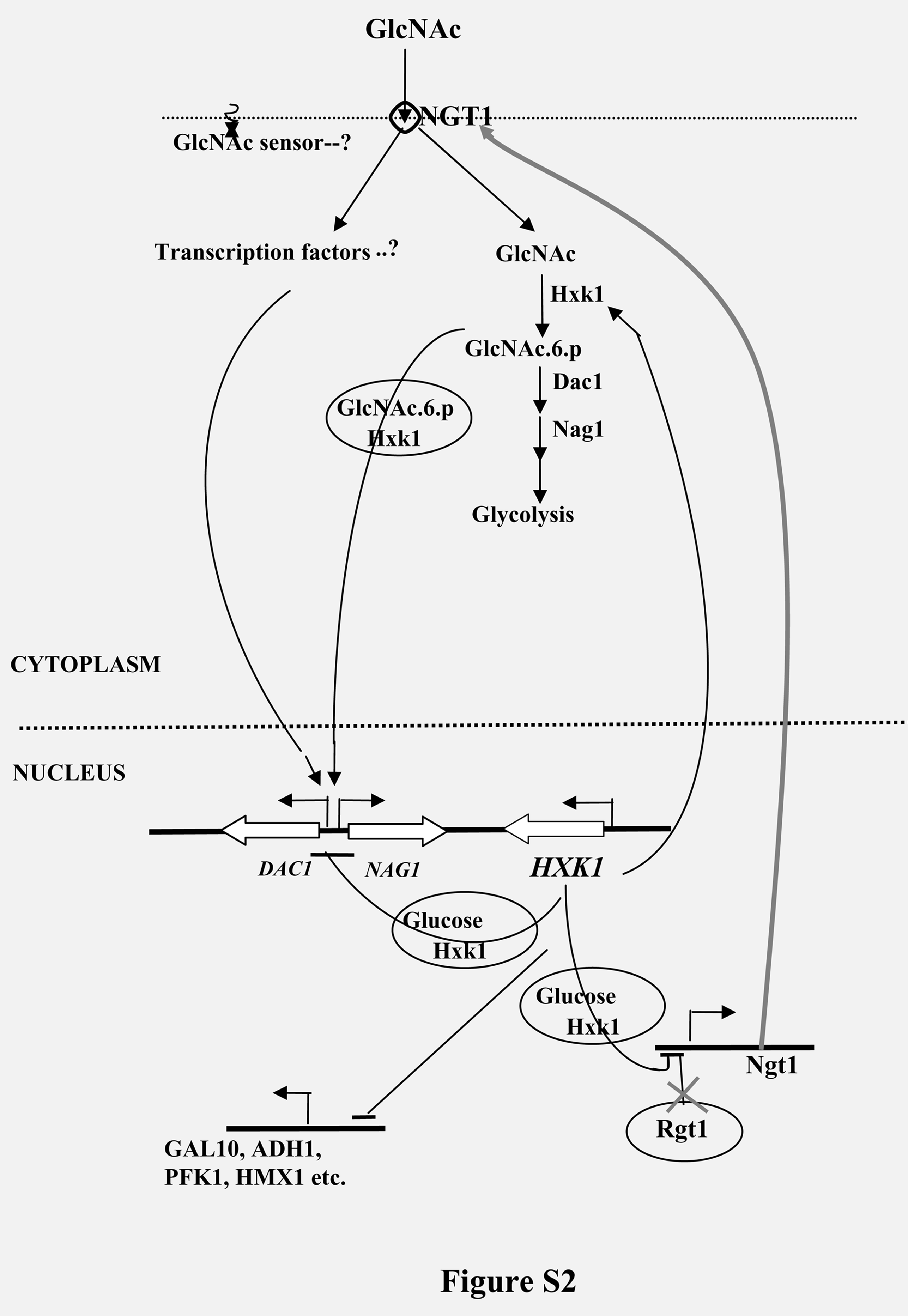

Supplement: Figure S2 — Mode of action of HXK1. Hxk1 plays a key role in the GlcNAc entry and GlcNAc induced gene expression. In the absence of glucose or other sugars, or presence of GlcNAc, NGT1 is relieved from Hxk1 repression (Fig. 9B); GlcNAc enters the cell and induces GlcNAc catabolic genes. In addition, freely entered GlcNAc is also able to induce gene expression as described in the recent literature by Naseem et al (2011). But, the GlcNAc sensor yet remains to be identified. Hxk1 also repress GAL10, GIG1, ADH1, PFK1 etc., There could be the probable involvement of uncharacterized regulator/s (GlcNAc also might directly interact with inducers to modulate the GlcNAc catabolic gene expression). (TIF) [file pone.0053638.s002.tif]

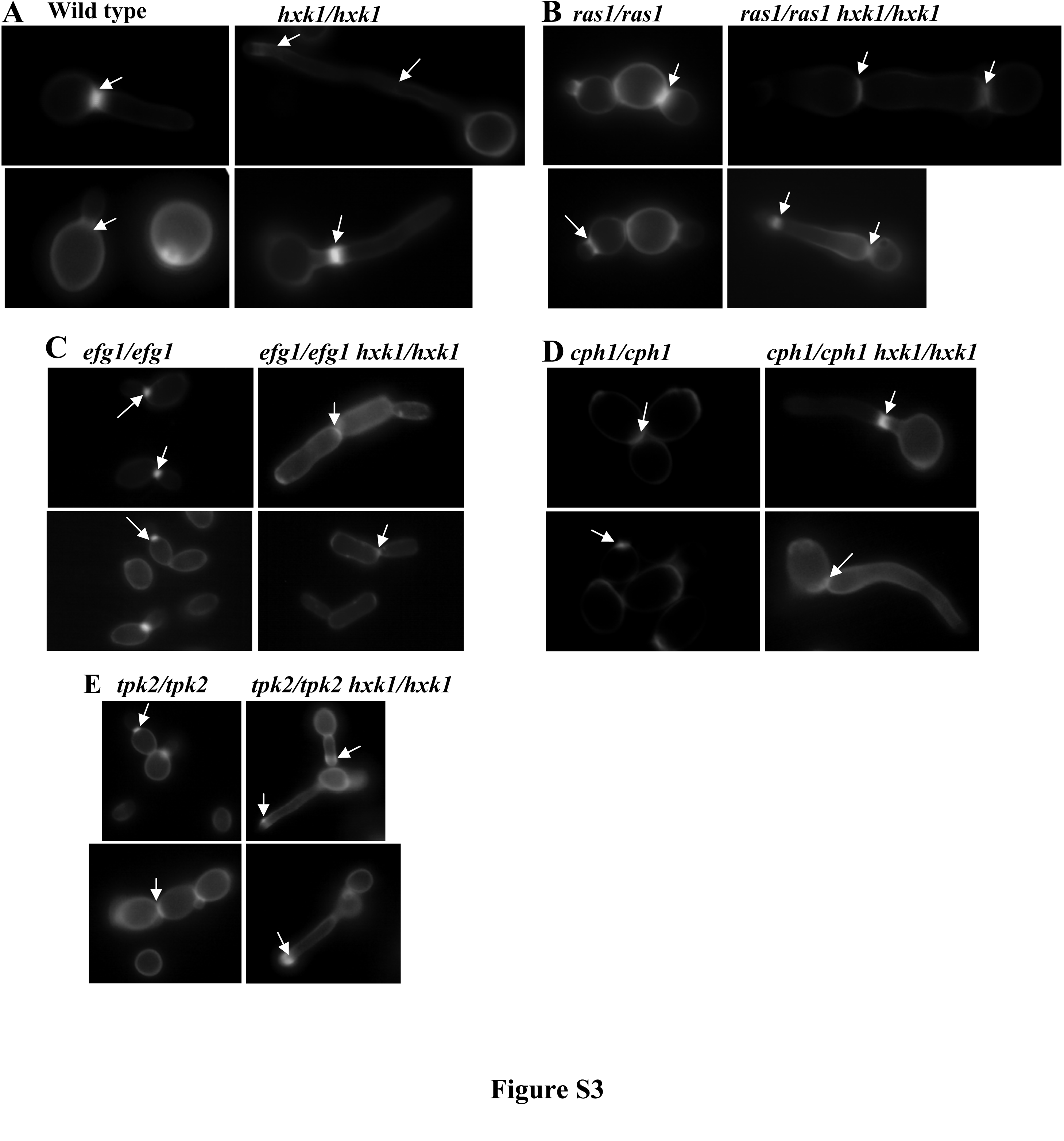

Supplement: Figure S3 — Calcofluor staining of hxk1 single and double mutants along with wild type. Cells were induced in Spider medium at 37°C for 2 hours and stained with Calcofluor white, which stains chitin in the cell walls and septa. White arrowheads show the septations. (TIF) [file pone.0053638.s003.tif]
