# Supplementary material for: N-Acetylglucosamine Kinase, HXK1 Is Involved in Morphogenetic Transition and Metabolic Gene Expression in Candida albicans
Source: PLoS One. 2013 Jan 14;8(1):e53638. doi: 10.1371/journal.pone.0053638 (PMC3544915; doi:10.1371/journal.pone.0053638)
Supplement: Table S1 — Quantitative estimation of filamentation in wild type, hxk1 single and double mutants. (DOC) [file pone.0053638.s004.doc]

**Table S1. Quantitative estimation of filamentation in wild type, *hxk1* single and double mutants.**

| Strain | Wild type | *hxk1* | *efg1* | *efg1hxk1* | *ras1* | *ras1hxk1* | *cph1* | *cph1hxk1* | *tpk2* | *tpk2hxk1* |
| --- | --- | --- | --- | --- | --- | --- | --- | --- | --- | --- |
| Germtube/  Filaments  (%) | 50-55 | 90-100 | 0 | 10-15 | 0 | 55-60 | 25-30 | 75-80 | 5-10 | 70 |
