# Supplementary material for: N-Acetylglucosamine Kinase, HXK1 Is Involved in Morphogenetic Transition and Metabolic Gene Expression in Candida albicans
Source: PLoS One. 2013 Jan 14;8(1):e53638. doi: 10.1371/journal.pone.0053638 (PMC3544915; doi:10.1371/journal.pone.0053638)
Supplement: Table S4 — Strains used in this study. (DOC) [file pone.0053638.s007.doc]

**Table S4. Strains used in this study.**

| **Strains** | **Relevant genotype and description** | **Source or reference** |
| --- | --- | --- |
| ***Candida albicans***  SC5314    CAI4  CAF2-1  H8-1-103  H8-1-103-R  iHXK1-GFP  iHXK1-Myc  iHXK1-HA  iHXK1-HF  BW-pYPB-HX-6HF-SIR-HA  BW-pYPB  CAI-pYPB-HX-HF  CAI-pYPB-HX-HF  CAN50  CAN52  HR1-4-2  HR1-4-2-R  HLC52  HLC67  HLC67-16-1-9  HLC67-16-1-9-R  A11-1  A11-1-1  AN8-1-16  AN8-1-16-R  TPO7.4    AS1  AS1-3-1-8  AS1-3-1-8-R  BCa2-9  BCa2-10  BCa2-9-H1  BCa2-9-R  SD  ***E.Coli* Srains**  DH5α | *URA3*/*URA3*  Δ*ura3*::*imm434*/Δ*ura3*::*imm434*  *Δura3*::*imm434/URA3*  *hxk1*Δ::*hisG/hxk1*Δ::*hisG-URA3-hisG*  *hxk1*Δ::*hisG/HXK1-URA3*  *CaHXK1-YFP1-ADH1T-URA3/CaHXK1*  *CaHXK1-13xMyc- ADH1T-URA3/CaHXK1*  *CaHXK1-3xHA-ADH1T-URA3/CaHXK1*  *TAP tagged HXK1expressed under ADH1 p in CAI-4*  *BWP17* transformed with pYPB-HXK.6HF plasmid and SIR2 tagged with 3ҳHA  *BWP17* transformed with pYPB-ADH1-pt plasmid  *CAI-4* transformed with pYPB-HXK.6HF plasmid  *CAI-4* transformed with pYPB-ADH1-pt plasmid  *ras1*Δ::*hisG/ras1*Δ::*hph-URA3-hph*  *ras1*Δ::*hisG/ras1*Δ::*hpn*  *ras1*Δ::*hisG/ras1*Δ::*hpn,hxk1*Δ::*hisG/hxk1*Δ::*hisG-URA3-hisG*  *ras1*Δ::*hisG/ras1*Δ::*hpn,hxk1*Δ::*hisG/HXK1-URA3*  *efg11*Δ::*hisG/efg11*Δ::*hisG-URA3-hisG*  *Δefg1::hisG/Δefg1;:hisGΔura3::imm434/Δura3*  *::imm434*  *Δefg1::hisG/Δefg1;:hisGΔura3::imm434/Δura3*  *::imm434, Δhxk1::hisG/Δhxk1::hisG-URA3-hisG*  *Δefg1::hisG/Δefg1;:hisGΔura3::imm434/Δura3*  *::imm434, Δhxk1::hisG/HXK1-URA3*  *cph11Δ::hisG/cph1Δ::hpn:hisG-URA3-hisG*  *Δcph1::hisG/Δcph1;:hisGΔura3::imm434/Δura3*  *::imm434*  *Δcph1::hisG/Δcph1;:hisGΔura3::imm434/Δura3*  *::imm434, Δhxk1::hisG/Δhxk1::hisG-URA3-hisG*  *Δcph1::hisG/Δcph1;:hisGΔura3::imm434/Δura3*  *::imm434, Δhxk1::hisG/HXK1-URA3*  *Tpk21Δ::hisG/tpk21Δ::hisG/hisG-URA3-hisG*  *Δtpk2::hisG/Δtpk2;:hisGΔura3::imm434/Δura3*  *::imm434*  *Δtpk2::hisG/Δtpk2;:hisGΔura3::imm434/Δura3*  *::imm434 Δhxk1::hisG/Δhxk1::hisG-URA3-hisG*  *Δtpk2::hisG/Δtpk2;:hisGΔura3::imm434/Δura3*  *::imm434 Δhxk1::hisG/HXK1-URA3*  *Δtup1::hisG/Δtup1;:hisGΔura3::imm434/Δura3*  *::imm434*  *Δtup1::hisG/Δtup1;:hisG-URA3-hisGΔura3::imm434/Δura3::imm434*  *Δtup1::hisG/Δtup1;:hisGΔura3::imm434/Δura3*  *::imm434, Δhxk1::hisG/Δhxk1::hisG-URA3-hisG*  *Δtup1::hisG/Δtup1:: hisG/hxk1Δ::hisG-URA3-hisGΔtup1::hisG/Δtup1;:hisGΔura3::imm434/Δura3*  *::imm434 Δhxk1::hisG/HXK1-URA3*  *ura3/ura3 sir2::dpl200/sir2 ::URA3-dpl200*  F’*/endA1 hsdR17*(r_K_^-^m_K_^+^) *gln*V44 *thi-1 rec*A1 *gyrA (Nal ^r^) relA Δ(laclZYA-argF)*U169 *deoR(φ80dlacΔ(lacZ)M15)* | W.A. Fonzi  W.A. Fonzi  W.A. Fonzi  This study  This study  This study  This study  This study  This study  This study  This study  This study  This study  Feng *et al*., 1999  Feng *et a*l., 1999  Laboratory strain  This study  G.R.Fink, 1997  G.R.Fink, 1997  Laboratory strain  This study  Laboratory strain  Laboratory strain  Laboratory strain  This study  J.F.Ernst., 2000  J.F.Ernst., 2000  Laboratory strain  This study  A.D.Johnson, 1997  A.D.Johnson, 1997  This study  This study  This study  Invitrogen Life Technology |
