# Supplementary material for: N-Acetylglucosamine Kinase, HXK1 Is Involved in Morphogenetic Transition and Metabolic Gene Expression in Candida albicans
Source: PLoS One. 2013 Jan 14;8(1):e53638. doi: 10.1371/journal.pone.0053638 (PMC3544915; doi:10.1371/journal.pone.0053638)
Supplement: Table S5 — List of primers used in the study. (DOC) [file pone.0053638.s008.doc]

**Table S5. List of primers used in the study:**

Primers used for *HXK1* disruption:

HBg 5’CCC ATA GAT CTA TGA CTG AGA CTA GC3’

HBX 5’CGT GTG AGA TCT CGA GTT CAT TAA TCG3’

HSp 5’GGA AAA GAC ATT TGC GGT GGC C3’

HPst 5’GGT CGA GTT GGT CGA AAG TAC C3’

Primers used for epitope tagging:

HX-F2

5’AggAggCCAAggggATAAAggTTgACTTgAAgTTgATTgAAAATgGCTCgATTATAggTgCTgccaTAggTgCTgCCTATCATAAg*CggATCCCCgggTTAATTAA* 3’

HX-UR-R1

5’ATAAgAgTTgAAgggATgTTACTCCAATTACACTAgTgCgATgTACACTTgggCTTgCTCATTCATCTTATATCATTTTATACATT*TCTAgAAggACCACCTTTgATTg* 3’

HX-GP-F1

5`AggAggCCAAggggATAAAggTTgACTTgAAgTTgATTgAAAATggCTCgATTATAggTgCTgCCATAggTgCTgCCTATCATAAgggTggTggT*TCTAAAggTgAAgAATTATT* 3’

HX-HFL-F

5`AggAggCCAAggggATAAAggTTgACTTgAAgTTgATTgAAAATggCTCgATTATAggTgCTgCCATAggTgCTgCCTATCATAA*gCTCgAgggTgCATgCCAC* 3’

HX-HFL-R

5’ATAAgAgTTgAAgggATgTTACTCCAATTACACTAgTgCgATgTACACTTgggCTTgCTCATTCATCTTATATCATTTTATACATT*gAgCggATAACAATTTCACACAgg 3’*

HX.AD.HF1 5’TATTATGACTGAGACTAGCATT 3’

HX.AD.R 5’GTTTATTTATCATCATCATCTTTA 3’

TAG1: 5’CGATTATTTCGATTAATGGAAC 3’

TAG2: 5’AGGAGGCCAAGGGGATAAA 3’

Primers used for probe DNA amplification:

HXK1 F 5’ATGACTGAGACTAGCATTAGTG3’

HXK1 R 5’CAACGAGTCGTTCAATATAG3’

RBT4 F 5’GTT GGT GCA ACT GTC ACT GG 3’

RBT4 R 5’GGG AGG AAG GTA GGA CAT GG 3’

HWP1 F 5’CAC TGT CCC ACA GGT AGA CG 3’

HWP1 R 5’GCA GGA GCT GAT GAT TTT GG 3’

ECE1F 5’CGC CCA CAA ATC TTA CAT ACA GG 3’

ECE1R 5’2 CCG GCA TCT CTT TTA ACT GG 3’

Primers used for Real-time PCR:

NGT1-RT-F 5’CGGCTCGTTGTTGTCATTCA 3’

NGT1-RT-R 5’CCTTGAGCTGCCCATAAAAGA 3’

NAG1-RT-F 5’AGGAGTGTGCCAACTACGAAAAG3’

NAG1-RT-R5’ CCCAACCCACCTAAGAACAAATC 3’

DAC1-RT-F 5’TCATAGCCACATCACCGTAA 3’

DAC1-RT-R 5’ATCGACGGGTCAACATGTACAC 3’

HWP-RT-F 5’ CGGAATCTAGTGCTGTCGTCTCT 3’

HWP-RT-R 5’ TAGGAGCGACACTTGAGTAATTGG 3’

RBT4-RT-F 5’ GTGGCTCCTCTTCTGGTAGTAATGA 3’

RBT4-RT-R 5’ CCATGTCTAGCACGTTTTTTGTTG 3’

ALS-RT-F 5’ CCAATGCTGCTAATTATGCTTTCA 3’

ALS-RT-R 5’ AACCCAAAACAGCATTCCAAGT 3’

ECE1-RT-F 5’ CATTCCACAAGTAATCCAAATCATCA 3’

ECE1-RT-R 5’ GACAACAAATGGCATATCAGCAA 3’

ACT1F 5’ ACGGTGAAGTTGCTGCTTTAGTT 3’

ACT1R 5’ GTCGTCACCGGAAAAACC3’

Primers used for *SIR2* deletion:

Sir del up F: 5’ ATATACGTTGGAGGTAAGCAAT 3’

Sir del up R: 5’ TATCCGCTCACAATTCCACATACTAGAGGATTTCTCTCAAATA 3’

Sir del down F: 5’ AAAAAGGGTCAGTGCTGCAATTAAATTAATATTGGTGTCTTTAAT 3’

Sir del down R: 5’ AAATTATCTTTTGATATCATTAGTG 3’

Primers for *SIR2* deletion confirmation:

CHEK-SIR 5’ AAAAAATAGGAATGTTGTTGAC 3’

CHEK-URA 5’GCTAATAAAGCTAATTACCTTG 3’

SIR-F 5’ ATGACAACTTTTTGGTCACAAAC 3’

SIR-R 5’GATATACGCCAACTCTTTTAAT 3’
