# Supplementary material for: N-Acetylglucosamine Kinase, HXK1 Is Involved in Morphogenetic Transition and Metabolic Gene Expression in Candida albicans
Source: PLoS One. 2013 Jan 14;8(1):e53638. doi: 10.1371/journal.pone.0053638 (PMC3544915; doi:10.1371/journal.pone.0053638)
Supplement: Text S1 — (DOC) [file pone.0053638.s009.doc]

**Supporting Text S1**

**Preparation of mutants**

The *HXK1* gene of *Candida albicans* (1.69kb) was amplified from genomic DNA using primers HBg (having a *Bgl*II site) and HBX (containing *Bgl*II and *Xho*I sites) and was initially cloned in pUC19 digested with P*vu*II and named as pHBgBx. 4.1kb *hisG-URA3-hisG* cassette released by *Sal*I-*Sac*I digestion of pUC19-CUB was ligated to the 3.443kb *Sal*I-*Sac*I (sites within the ORF) digested vector backbone of pHBgBX to generate the plasmid pHxk-B. The *ura* minus strain of wild type *C.albicans*, *CAI4* was transformed with *Bgl*II digested pHxk-B plasmid by lithium-acetate method.

The Ura positive transformants obtained after 2 days were checked by Southern and were selected for curing of the *URA* marker. A similar round of transformation with pHXK-B plasmid was carried using the *URA* cured colony for the second allele disruption. The colonies obtained were screened by Southern and one such colony H8-1-103 was selected for further studies. The strain H8-1-103 was *URA* cured by a similar procedure described above.

For complementation, the full length *HXK1* gene was cloned and integrated to the null mutant at one of the disrupted alleles. For this purpose,the *hxk1* double mutant H8-1-103-4 was transformed with the 5kb *Pst*I- *Sph*I fragment of pHXK-R (plasmid containing the 2.3 kb*URA3* fragment obtained by digesting pUC19-CUB and ligating to a backbone of pHXK-I that contains a full-length *HXK1* using Primers HSp and HPst). The positive clone having HXK1-URA3 insertion in one of the two alleles was selected for further analysis and was named as H8-1-103-4-P1. For disrupting both the alleles of *HXK1* in other filamentous specific mutants (*ras1*, *efg1*, *cph1*, *tpk2*, *tup1*) a similar procedure as described above was followed.

**Preparation of *sir2* mutant**

The *sir2* mutant strain, SD was constructed in *C. albicans* strain *CAI4* background using the PCR fusion strategy (1) and *URA3-dpl200* cassette (2) by electroporation (3). 625 bp up-stream and 556 bp down-stream regions of *SIR2* gene were amplified by keeping 20 bp homology (bold letters in the primer sequence) to *URA3-dp*l200 cassette ( primer pairs: Sir del up F and Sir del up R., Sir del down F and Sir del down R). In the next round of amplification the above mentioned fragments used as primers and pDBB57 plasmid which houses the URA3-dpl200 cassette acted as template. Two rounds transformation and *URA3* curing was carried out as mentioned in *hxk1* mutant preparation. Correct integration was confirmed by PCR ( primers: CHEK-SIR, CHEK-URA and the absence of the *SIR2* ORF in strain SD was confirmed by PCR ( primers: SIR-F, SIR-R).

**Supporting References :**

S1. Wach A **(**1996) PCR-synthesis of marker cassettes with long flanking homology regions for gene disruptions in S. cerevisiae. Yeast 12**:** 259–265

S2**.** Wilson RB, DDavis BM, Enloe, AP Mitchell **(**2000) A recyclable Candida albicans URA3 cassette for PCR product-directed gene disruptions. Yeast 16: 65–70.

S3. Reuss O, Vik A, Kolter R, Morschhauser J (2004) The SAT1 flipper, an optimized tool for gene disruption in Candida albicans. *Gene* 341, 119–127
